# Improvement of Gold Nanorods in Photothermal Therapy: Recent Progress and Perspective

**DOI:** 10.3389/fphar.2021.664123

**Published:** 2021-04-22

**Authors:** Shengnan Liao, Wang Yue, Shuning Cai, Quan Tang, Weitong Lu, Lingxiao Huang, Tingting Qi, Jinfeng Liao

**Affiliations:** ^1^ State Key Laboratory of Oral Diseases, National Clinical Research Centre for Oral Diseases, West China Hospital of Stomatology, Sichuan University, Chengdu, China; ^2^ Department of Radiation Biology, Radiation Oncology Key Laboratory of Sichuan Province, Department of Clinical Pharmacy, Sichuan Cancer Hospital and Institute, Sichuan Cancer Center, School of Medicine, University of Electronic Science and Technology of China, Chengdu, China; ^3^ State Key Laboratory of Biotherapy and Cancer Center, West China Hospital, Sichuan University, Collaborative Innovation Center of Biotherapy, Chengdu, China

**Keywords:** Cancer, Photothermal therapy, Localized tumor treatment, Gold nanorods, Nanomaterials

## Abstract

Cancer is a life-threatening disease, and there is a significant need for novel technologies to treat cancer with an effective outcome and low toxicity. Photothermal therapy (PTT) is a noninvasive therapeutic tool that transports nanomaterials into tumors, absorbing light energy and converting it into heat, thus killing tumor cells. Gold nanorods (GNRs) have attracted widespread attention in recent years due to their unique optical and electronic properties and potential applications in biological imaging, molecular detection, and drug delivery, especially in the PTT of cancer and other diseases. This review summarizes the recent progress in the synthesis methods and surface functionalization of GNRs for PTT. The current major synthetic methods of GNRs and recently improved measures to reduce toxicity, increase yield, and control particle size and shape are first introduced, followed by various surface functionalization approaches to construct a controlled drug release system, increase cell uptake, and improve pharmacokinetics and tumor-targeting effect, thus enhancing the photothermal effect of killing the tumor. Finally, a brief outlook for the future development of GNRs modification and functionalization in PTT is proposed.

## Introduction

According to the Global Cancer Observatory, cancer is one of the most life-threatening diseases because of its increasingly higher incidence and death rate (Bray et al., 2018). However, existing therapeutic methods toward cancer, which chiefly include chemotherapy, radiotherapy (RT), and surgical treatment, have inevitable shortcomings in clinical practice (Akhter et al., 2018). For example, the therapeutic effect of chemotherapy can be unsatisfactory considering the heterogeneity of tumors and its potential systemic toxicity due to poor targeting selectivity. Similarly, RT could have a negative impact on the patients’ immunological function and blood system, causing immune dysfunction, and the rapid spreading of cancer cells throughout the body. The lack of specific tumor-targeting effects is a quite knotty problem all the time (Hughes 2003; Wistuba et al., 2011; Lee et al., 2012). Photothermal therapy (PTT), also known as photothermal ablation, is a relatively new type of cancer treatment that usually utilizes the injection of a material with high photothermal conversion efficiency. These nanomaterials possess the ability to gather near the tumor tissue and subsequently be exposed to heat-generating near-infrared (NIR) light to kill cancer cells under hyperthermal environments (Hussein et al., 2018). The emergence of PTT in 2000 provided a potential countermeasure to cancer. Compared to traditional therapeutic methods, PTT realized the tumor-specific treatment by focusing the optical irradiation on the tumor site, which can dramatically increase the tumor-killing efficiency and reduce systemic toxicity.

**FIGURE 1:**
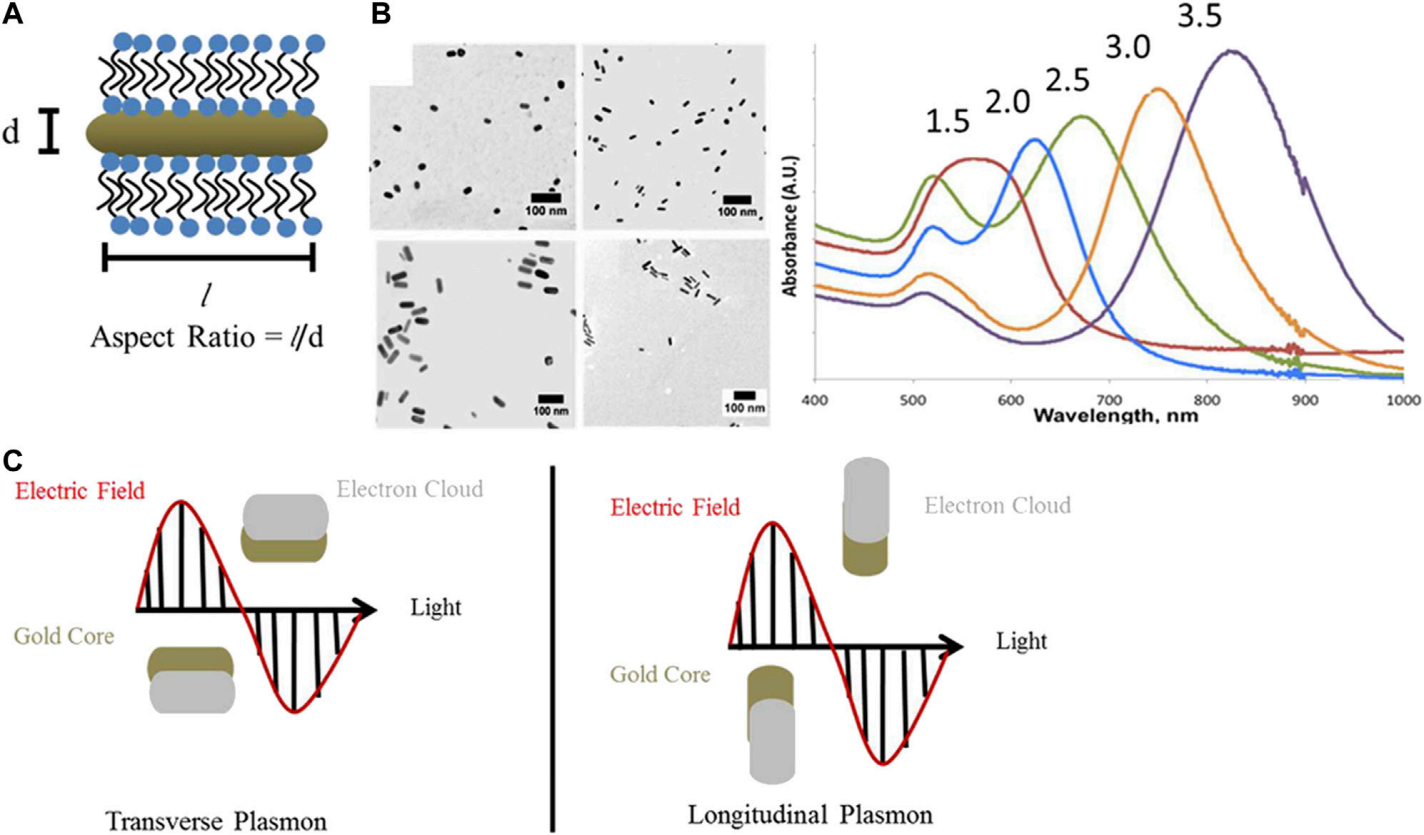
Optical properties of gold nanorods. **(A)** GNRs have two surface plasmon resonance bands: one transverse and the other longitudinal. The longitudinal one varies with the aspect ratios (AR). Aspect Ratio = l/d. **(B)** TEM images and UV−vis−NIR extinction spectra of GNRs with AR 1.5–3.5, aspect ratios are indicated above the absorbance spectrum of each sample. Scale bars are 100 nm. **(C)** Schematic representation of transverse and longitudinal plasmon absorbances in GNRs (Lohse and Murphy 2013).

Photothermal agents used to mediate PTT are constituted by inorganic and organic nanomaterials. Inorganic nanomaterials mainly include precious metal nanoparticles [gold, silver (Boca et al., 2011), platinum (Manikandan et al., 2013), and palladium (Zhou et al., 2012) nanoparticles], carbon-based nanomaterials [carbon nanotubes (Kam et al., 2005) and graphene (Su et al., 2016)], and metal chalcogenides [such as copper sulfide (Li et al., 2010)]. Organic nanomaterials chiefly include NIR dyes [indocyanine green (Zheng et al., 2011), Prussian blue (Fu et al., 2012), etc.] and conjugated polymers (Yang et al., 2011). Among them, gold nanoparticles with different shapes, owing to their unique optical-electron properties, have received widespread attention in PTT (Young et al., 2012). Under a particular frequency of incident light, free electrons resonate at the metal’s surface and reach the maximal amplitude of oscillation, termed surface plasmon resonance (SPR). The oscillation nonradiative decay, converting light energy to heat, thus can induce strong light absorption and provide higher photothermal conversion efficiency (Liu et al., 2018). Rod-shaped gold nanoparticles, also known as gold nanorods (GNRs), are anisotropic and tunable, which means that their optical and chemical properties alter with directions and synthesis parameters. For instance, GNRs have two SPR bands, including the longitudinal one that varies with the aspect ratio (AR; longitude/transverse; Onaciu et al., 2019; Takahata et al., 2018) and the transverse one that is relatively constant (Figure 1; Haine and Niidome 2017; Lohse and Murphy 2013). GNRs show excellent light absorption in the visible-NIR spectral region and the longitudinal peak redshifts as the AR increases (Kennedy et al., 2011). The incident light with wavelengths in the NIR region (650–1100 nm) is usually chosen because it demonstrates deep penetration into the body and is rarely absorbed and scattered before reaching the GNRs (Hwang et al., 2014; Bhana et al., 2016). Irradiating GNRs with NIR light produces the moderate temperature rise in the target region that is needed to selectively damage tumor tissues, which are more sensitive to hyperthermia than healthy tissues.

GNRs can be synthesized by multiple methods, including template synthesis (Wirtz et al., 2002), photochemical synthesis (Xu et al., 2016), electrochemical synthesis (Huang et al., 2006), wet chemical seed-mediated synthesis (Jana et al., 2001), and seedless method (Liu X. et al., 2017), all of which are reproducible and controllable. At present, seed-mediated synthesis using small nanoparticles or short nanorods as seeds, and adding HAuCl_4_ to develop GNRs with adjustable ARs under the help of a surfactant, usually hexadecyltrimethylammonium bromide, is the most mature method (Xia et al., 2015). Up to now, GNRs are one of the most commonly studied materials for potential biomedical applications, including bioimaging (Li P. et al., 2018), molecular detection (Kwizera et al., 2018), drug delivery (Hossen et al., 2019), and PTT (Zhang et al., 2018). Despite the advantages mentioned above, GNRs can be further improved. Recently, there is a vast amount of research focusing on the optimization of GNRs in several aspects, including size, surface functionalization, and the expansion of production. There is still room for improvement of its efficiency in PTT. For in-depth comprehension and practical application, more *in vitro* and *in vivo* studies are needed. Besides, the combination of GNRs-mediated PTT with other therapies, such as chemotherapy, gene therapy, and immunotherapy, also makes a significant difference (Riley and Day 2017).

This review mainly covers the synthesis methods of GNRs, their surface functionalization, and applications in tumor therapy. The common synthesis methods are first introduced, followed by the measures to reduce the toxicity, increase the yield, and control the size and shape. Then, different GNRs-based drug loading and controlled release systems are introduced, as well as the surface functionalization measures in improving the *in vitro* uptake rate of cells, prolonging blood circulation, increasing tumor accumulation, and enhancing the efficiency of PTT. Finally, the shortcomings and further developments of these studies are pointed out, providing a broader idea for prospective PTT based on GNRs.

## Synthesis Methods of GNRs and Their Improvements

In the past decade, research on the application of GNRs has been increasing, mainly focusing on biological imaging, PTT, and other fields. With the gradual development of the study, the difference in the size and crystal structure of GNRs has a significant effect on PTT, and the effective regulation of the synthesis of GNRs directly determines its structure and subsequent application. Up to now, there is still a lack of deep understanding of the exact mechanism of morphology and size control in the growth process of GNRs, and it is always at the forefront of current research. The synthesis methods of GNRs mainly include the template method, electrochemical synthesis method, seed-mediated growth method, and seedless synthesis method. Here, the improvement of preparing GNRs by seed-mediated growth method is mainly introduced (Table 1).

**TABLE 1:** Summaries of synthesis methods of gold nanorods and their improvements.

Synthesis methods	Goals of improvements		Specific methods	References
Seed-mediated growth method	Reduce toxicity		A simple “one-pot method” was proposed which adds sodium borohydride to remove CTAB.	He et al. (2018)
			Replacing ascorbic acid with dopamine	Requejo et al. (2017)
			Using a less toxic surfactant, dodecyl dimethyl ammonium bromide (C12EDMAB) as an alternative	Allen et al. (2017)
			Synthesizing hollow GNRs with nontoxic modifiers	Cai et al. (2018)
	Control the AR		Increasing the concentration of silver nitrate for higher AR	Su et al. (2015); Gallina et al. (2016)
			Adjusting the reaction time	Zhang et al. (2017b)
			Adjusting the temperature	Liu et al. (2017b)
			Adjusting the pH	Zhang et al. (2014a); (Chang and Murphy 2018)
			Adjusting the concentration of the ascorbic acid	Li et al. (2018a)
			Adjusting the amount of the seeds	Su et al. (2015)
			Adjusting the concentration of the CTAB.	Hormozi-Nezhad et al. (2013)
			Using 3-aminophenol as the reducing agent	Wu et al. (2019b)
			Adding HCl	Wang et al. (2016c)
			Adding bioadditives like glutathione or small thiolated molecules	Requejo et al. (2020)
			Thermal reshaping	Huang et al. (2018)
	Control the size		Adjusting the concentration of seeds added in the growth solution	(Chang and Murphy 2018); Cheng et al. (2019); Mbalaha et al. (2019)
	Control the end shape		Longer cylindrical-shaped GNRs were synthesized by adding HCl and dog-bone shaped GNRs were fabricated in the group without HCl	Wang et al. (2016c)
			The number of anions rather than the pH altered by HCl chiefly determines the end shape	Kim et al. (2016)
	Improve the monodispersity		Replacing the ascorbic acid with hydroquinone	Ghosh et al. (2017)
			Replacing the ascorbic acid with 3-aminophenol	(Vigderman and Zubarev 2013)
			Replacing the ascorbic acid with pyrogallol	Huang et al. (2015)
			Replacing the ascorbic acid with dopamine	Su et al. (2015)
	Improve the reproducibility		Continuous agitation at a constant temperature of 30°C	Gallina et al. (2016)
			Using CTAB and n-decanol as surfactants Seperating the symmetry breaking and the seeded growth process	Gonzalez-Rubio et al. (2019)
			Secondary growth of GNRs	Khlebtsov et al. (2014a); Khlebtsov et al. (2014b)
			Controlled etching	Szychowski et al. (2018)
	Improve the yield		Increasing HAuCl_4_ concentration and slowly adding ascorbic acid	(Khanal and Zubarev 2019)
			Simultaneously increasing the concentration of seeds and reactants	Park et al. (2017)
			Adding ascorbic acid to GNRs solution continuously	Kozek et al. (2013)
			Replacing ascorbic acid with hydroquinone	Requejo et al. (2018)
			Using hydroquinone as a reducing agent	Zhang et al. (2014a)
			Using 3-aminophenol as the reductant	Wu et al. (2019b)
			Introducing sliver ion for high yields of short GNRs	Hormozi-Nezhad et al. (2013)
			Using freshly prepared silver nitrate and ascorbic acid solutions	Burrows et al. (2017)
			Using different concentrations of GSSG at 30 min of reaction	Requejo et al. (2020)
			Raising pH	Xia et al. (2015)
			Asymmetric-flow field flow fractionation (A4F)	Nguyen et al. (2016)
			Introducing the right proportion of seeds, Au^3+^ ion, ascorbic acid, and CTAB	(Sau and Murphy 2004)
Seedless method	Improve morphology		Adjusting the concentrations of CTAB and sodium oleate	Roach et al. (2018)
	Promote the anisotropic growth		Introducing a weaker reducing agent (hydroquinone), with template modification	Liu et al. (2017a)
			Introducing a weaker reducing agent (resveratrol)	Wang et al. (2016b)
	Improve the yield		Increasing the concentration of the gold precursor solution	Yan et al. (2018)

### Seed-Mediated Growth Method

Since the first approach of GNRs, the synthesis methods of GNRs have been greatly developed (Lohse and Murphy 2013). Seed-mediated synthesis is currently the most commonly used method, which is to add a certain amount of gold nanoparticle seeds into the growth solution followed by the growth of seeds into GNRs with the help of surfactants. Jana et al. were the first to synthesize GNRs using the seed-mediated growth method (Jana et al., 2001). The process can be divided into three steps: 1) citrate-capped gold nanospheres used as seeds are formed after the reduction of HAuCl_4_ by NaBH_4_, 2) the growth solution that contained HAuCl_4_ and cetyltrimethylammonium bromide (CTAB) is prepared, and 3) GNRs are acquired by adding seeds to the growth solution and ascorbic acid (AA; Jana et al., 2001; Xia et al., 2015). However, the low yield and unsatisfied size of GNRs synthesized in this original way make it inappropriate for applications (Scarabelli et al., 2015; Allen et al., 2017), so there appears an endless stream of improvements in accordance with the requirements based on GNRs applications in PTT.

GNRs suitable for PTT require good monodispersity, small size (An et al., 2017; Cheng et al., 2019), anisotropy, no toxicity, and the need to be produced in high yields. To obtain ideal GNRs, previous studies have discussed the influence of various factors on the synthesis process (Scarabelli et al., 2015) and adopted different improvement methods, including silver-assisted seeded growth (Nikoobakht and El-Sayed 2003), using CTAB-capped seeds instead of citrate-capped ones, etc., which can help grow GNRs to the desired length (He et al., 2017). The improved methods are not limited to this. Various improved methods follow, aiming at obtaining GNRs with better biocompatibility to guarantee safety and better controlling the AR, size, and rod shape.

#### Reduce Toxicity

At present, CTAB is usually introduced as a surfactant in the seed synthesis method of GNRs and is an indispensable step in the seed synthesis method. The CTAB concentration is closely related to the yield, shape, and size of GNRs. Studies have shown that CTAB as a surfactant can prevent isotropic grain growth, thus preventing it from forming spherical by-products (Mbalaha et al., 2019). CTAB of different suppliers can lead to different synthesis results of GNRs (Scarabelli et al., 2015). However, it is worth noting that CTAB molecules remaining on both the suspension solution and the GNRs surface are identified as the source of cytotoxicity. CTAB can cause damage to mitochondria and induce apoptosis by entering cells with or without GNRs. Therefore, how to effectively control the toxicity of surfactant CTAB during the preparation of GNRs is an urgent problem to be solved (Qiu et al., 2010; Golubev et al., 2016). During the production process, the toxic effects of CTAB can be reduced by repeated cleaning and replacement of nontoxic modifiers. Several protocols have emerged to remove CTAB from the surface of GNRs in previous studies, but most of them require tedious steps and costly reagents. He et al. proposed a simple “one-pot method” to completely remove CTAB from the surface of GNRs. This procedure adds sodium borohydride to remove CTAB as efficiently as the commercially available GNRs sample (He et al., 2018). Studies have shown that replacing AA with dopamine can also reduce the concentration of CTAB (Requejo et al., 2017).

Besides removing CTAB as much as possible to reduce its concentration, switching to other nontoxic surfactants is also an excellent way to reduce the toxicity of GNRs. Xu, Blahove et al. led the synthesis of GNRs with less toxicity using a less toxic surfactant, dodecyl dimethyl ammonium bromide (C12edmab), as an alternative (Allen et al., 2017). Hollow GNRs with controllable AR were synthesized by Cai et al. with nontoxic modifiers, which also reduced toxicity (Cai et al., 2018). Although the above methods reduce the toxicity of residual CTAB to some extent, how to develop a more simple and convenient method to reduce toxicity is still worthy of further studies. Above all, how to choose a nontoxic synthesis method or material to replace CTAB, which can be produced on a large scale and pass clinical trials, is a key and challenging point. The method described here to reduce the toxicity of GNRs is only aimed at the synthesis process of GNRs themselves, and it will be mentioned later on how to conceal the residual CTAB by surface functionalization or substitutions on GNRs.

#### Control the Size and Shape

GNRs with distinct sizes, ARs, and end shapes have been designed for specific uses. First, studies on the factors affecting the ARs of GNRs are condemned to be valuable, as longitudinal SPR (LSPR), which influences the optical properties of GNRs dramatically, can be finely tuned by adjusting the ARs. Numerous studies have demonstrated that GNRs with high ARs could be obtained by increasing the concentration of silver nitrate in the process of silver-assisted seed-mediated synthesis (Su et al., 2015; Gallina et al., 2016). Tong et al. found that silver nitrate plays a vital role in the symmetry-breaking point, and it was the [HAuCl_4_]/[AgNO_3_] ratio in the growth solution that critically controls the final width of GNRs and thus the ARs (Tong et al., 2017). Other factors, through the synthesis, such as reaction time (Zhang J. et al., 2017), temperature (Liu et al., 2017c), pH (Zhang L. et al., 2014; Chang and Murphy 2018), the concentration of AA (Li P. et al., 2018), seeds (Su et al., 2015), CTAB (Hormozi-Nezhad et al., 2013), types of reductants (Wu Z. et al., 2019), and surfactants and additives (Wang Y. et al., 2016), also have a great influence. Recently, Requejo et al. have proven that the addition of bioadditives, such as glutathione (GSH) or small thiolated molecules, in nanomolar and micromolar concentrations during the growth stage facilitated the formation of GNRs with tunable ARs and LSPR (Requejo et al., 2020). Additionally, more techniques, such as thermal reshaping, have been gradually used in the production of AR-tuned GNRs (Huang et al., 2018).

The plasmonic properties of GNRs also depend on specific sizes. Larger GNRs represent the higher scattering/absorption ratio, allowing scattering-based applications, such as imaging, whereas smaller GNRs exhibit great potential in PTT toward tumors due to their comparably larger absorption cross-sections. However, traditional synthesis methods of ultrasmall GNRs, by increasing the concentration of seeds added in the growth solution, show a lot of inevitable problems, such as the decreased growth yield and the more by-products, such as nanospheres (Tatini et al., 2014; Chang and Murphy 2018; Cheng et al., 2019; Mbalaha et al., 2019). Therefore, new synthesis strategies of small-sized GNRs should be put forward as soon as possible. Some researchers have attempted to explore the relationship between different end shapes of GNRs and their SPR effects. Wang et al. demonstrated that the addition of hydrochloric acid (HCl) successfully slowed down the growth rate of GNRs, creating longer cylindrical GNRs, whereas short, dogbone-shaped GNRs were fabricated in the group without HCl (Wang Y. et al., 2016). Interestingly, in another research, an inconsistent phenomenon was observed that the end shape of GNRs changed from an arrowhead shape to a dumbbell-like and a dog bone-like shape as the concentration of HCl increased through the second growth of GNRs. The subsequent results showed it was the number of anions rather than the pH altered by HCl that chiefly worked in this process (Kim et al., 2016).

Apart from the specific size, AR, and end shape, the ideal synthetic product of GNRs should guarantee the superior monodispersity and reproducibility of the synthesis. Many studies have synthesized GNRs with quite narrow size distribution and high shape purity by replacing the AA with weaker reductants, such as hydroquinone (Ghosh et al., 2017), 3-aminophenol (Vigderman and Zubarev 2013), pyrogallol (Huang et al., 2015), dopamine (Su et al., 2015), etc. However, good reproducibility remains an unattainable goal for the lack of understanding of the fabrication mechanism at a molecular level (Gallina et al., 2016). Improved reproducibility was achieved through continuous agitation and at a constant temperature of 30°C, which was believed to guarantee the complete solubilization of CTAB, by Gallina et al. (2016). Poor reproducibility is believed to be associated with the stochastic nature of the symmetry-breaking event, which plays a critical role in the subsequent anisotropic growth process during the synthesis of GNRs (Walsh et al., 2017b). Thus, Gonzalez-Rubio et al. attempted to separate the symmetry-breaking step from the seeded growth process. n-Decanol was addicted to the surfactant CTAB to generate a micellar aggregate. They first prepared the intermediate anisotropic seeds (small GNRs) with smaller dispersions in size and shape and subsequently induced GNRs based on them with specific AR and size by controlling the pH, temperature, and Ag^+^ concentration. GNRs with LSPR bands ranging from 600 to 1270 nm are accessible by this method without significantly affecting their dispersion in size and shape, which simultaneously optimize the symmetry breaking and the seeded growth process (Gonzalez-Rubio et al., 2019). Besides, postsynthesis modification, including the secondary growth (Ratto et al., 2010; Khlebtsov et al., 2014a; 2014b) and controlled etching (Szychowski et al., 2018) of GNRs, serves as an effective strategy to improve the reproducibility and precisely control the shape of GNRs.

Although research on the synthesis of various shapes of GNRs has made progress, there are still some problems that have not been solved, such as the limitations of the existing synthesis method of small-sized GNRs, the lack of understanding of the underlying molecular mechanisms in the growth of GNRs, the unattainable reproducibility of the synthesis, etc.

#### Improve the Yield

The conversion of Au salt precursor into GNRs contains two main parts. One is the reduction of Au [3] into Au [0] and the other is the formation of nanorods (Park et al., 2017). Various methods have been used to improve the yield of GNRs, involving increasing the total amount of Au0 in suspension, improving the ratio of GNRs to by-products, and enhancing the monodispersity of the GNRs and for the ultimate goal of expanding the production scale.

To raise the yield of reduced Au, Kozek et al., and Ratto et al. reported a secondary growth process in which AA was continuously added to deposit Au precursor remaining in the solution on GNRs (Ratto et al., 2010; Kozek et al., 2013). Although AA is critical for the growth of GNRs, a high concentration of it is accompanied by increasing by-products. Therefore, different reactants are added to decrease the side products. Some of them are weaker reducing agents, such as hydroquinone (Zhang L. et al., 2014; Chang and Murphy 2018; Requejo et al., 2018) and 3-aminophenol (Wu Z. et al., 2019) instead of AA. The addition of silver ions helps cut down the percentage of spherical nanoparticles in the product and form stable GNRs (Hormozi-Nezhad et al., 2013; Jessl et al., 2018), but Ag^+^ needs to be freshly prepared like AA (Burrows et al., 2017). Seeds, Au [3], AA, and CTAB in the correct proportion are necessary for producing high-yield GNRs (Sau and Murphy 2004). The purity of CTAB, mainly affected by bromide ion, also influences the yield of GNRs (Rayavarapu et al., 2010; Si et al., 2012). The reaction conditions and separation methods are the prime factors that affect the yield, too. Adding sodium hydroxide raises the pH value, resulting in an increase in the yield of rod-shaped nanoparticles (Xia et al., 2015), whereas adding HCl retards the growth of GNRs (Wang Y. et al., 2016). Surfactants could be used to preliminarily assist the precipitation of gold nanoparticles of different shapes in a concentrated dispersion (Xia et al., 2015). To extract pure nanorods, Nguyen et al. separated seeds and by-products by asymmetric-flow field flow fractionation (A4F) and finally increased the output of GNRs (Nguyen et al., 2016). Besides, it is necessary to improve resource utilization efficacy and simplify purification steps (Park et al., 2017).

### Seedless Synthesis Method

Different from the seed-mediated growth method, the seedless method does not require a separate seed solution but directly adds sodium borohydride to the growth solution, which can directly reduce Au^3+^ to Au to form seeds due to its strong reducibility. Then, the gold ion in the solution is slowly reduced under the action of weak reducing agents, grows along with the seed longitudinally under the mediation of CTAB, and finally forms a rod-like structure (An et al., 2017). The seedless method is often utilized to synthesize GNRs of small size (<5 nm in diameter) due to the tiny seed formed directly in the growth solution. The size of GNRs decreases in inverse proportion to the concentration of sodium borohydride added. To be more specific, a higher concentration of sodium borohydride leads to an increased amount of gold nuclei. As the total concentration of Au in the solution is constant, the number of gold atoms deposited on each gold nucleus decreases, resulting in the reduction of the final size of GNRs (Tatini et al., 2014).

Jana first discovered the seedless method, but the resulting product has poor monodispersity and more spherical by-products (Jana 2005). EL-Sayed optimized the pH value and sodium borohydride concentration based on Jana, thus improving the monodispersity and yield of the product, changing the AR of GNRs (Ali et al., 2012). There have been more and more optimization measures for seedless methods in recent years, and the quality of synthesized products has gradually increased. Lai et al. added sodium oleate to the solution, which not only expanded the diameter range but also achieved high yield and high monodispersity (Lai et al., 2014). Based on Lai et al., Lucien et al. simultaneously manipulated the concentrations of CTAB and sodium oleate to achieve effective control of the morphology of GNRs (Roach et al., 2018).

Liu et al. replaced AA with a weaker reducing agent (hydroquinone) and cooperated with template modification, which greatly promotes the anisotropic growth of GNRs (Liu K. et al., 2017). Wang et al. used resveratrol as a reducing agent. Its weak reducibility can not only avoid secondary nucleation but also affect the adsorption of CTAB, thereby promoting the anisotropic growth of crystal grains (Wang W. et al., 2016). Other surface functionalization measures include the following: Yan et al. increased the concentration of the gold precursor solution to achieve a larger-scale synthesis of high-quality small GNRs (Yan et al., 2018). Katherinne et al. discovered that the addition of bioadditives, such as GSH and oxidized GSH (GSSG), to the solution could cause different effects on the AR, size, and yield of GNRs (Requejo et al., 2020).

## Functionalization Aimed at Specific Procedures of GNRs for PTT

Generally speaking, the PTT effect of nanomaterials after entering the body is a continuous process, which includes retention in the blood circulation, interaction with the tumor microenvironment (TME), and ingestion by tumor cells to exert the PTT therapy effect. These links are inseparable, and each plays an essential role in killing tumors by materials based on GNRs. Some recent improvements for each specific step in PTT will be put forward, opening up a broader clinical prospect for the materials based on GNRs (Table 2).

**TABLE 2:** Summaries of functionalization of gold nanorods aimed at specific procedures of photothermal therapy.

The specific procedure	Materials	Therapy type	Cell line	Cancer model	References
Improve cellular uptake efficiency	PEG-coated and DNA-coated GNRs	-	-	-	Yang et al. (2016a)
	CTAB-coated GNRs, polystyrene sulfonate (PSS)-coated GNRs, and poly (diallyldimethyl ammonium chloride)(PDDAC)-coated GNRs	-	-	-	Sun et al. (2018)
	Citrate acid stabilized GNRs and transferrin-coated GNRs	-	HeLa	-	Chithrani et al. (2006)
	GNRs that feature cationic ligands with diverse headgroups	-	HeLa	-	Saha et al. (2013)
	GNRs functionalized with hairpin DNA (hpDNA)	-	HeLa	-	Zhang et al. (2015)
	Folate-functionalized silica-coated GNRs	-	HepG2	Rabbit liver VX-2 tumor	Gao et al. (2016)
	Neutral and cationic PEG-decorated GNRs	-	-	-	Mahmoud et al. (2019b)
	Anionic poly acrylic acid (PAA)-decorated GNRs and bovine serum albumin (BSA)-coated GNRs				
	Herceptin–GNRs complexes	Chemotherapy	SK-BR-3	-	Jiang et al. (2008)
	PEGylated GNRs	-	HeLa	-	Abdelrasoul et al. (2016)
	Phospholipid-PEG-GNRs	Chemotherapy	MCF-7	-	Mahmoud et al. (2019a)
			T47D		
	Chitosan-capped GNRs	-	HepG2	-	Lee et al. (2019)
	CTAB-coated GNRs and polyelectrolyte-coated GNRs	Chemotherapy	MCF-7	-	Qiu et al. (2010)
	Polyelectrolyte-coated GNRs and PEG-GNRs	-	-	-	Alkilany et al. (2012)
	GNRs coated with (16-mercaptohexadecyl) trimethylammonium bromide (MTABGNRs)	PTT	DU145	-	Zarska et al. (2018)
			HeLa		
			TRAMP-C2		
	GNRs coated with CTAB, polyoxyethylene cetyl ether, oligofectamine, and phosphatidylserine	-	-	-	Kah et al. (2014)
	CTAB capped gold nanorods, GNRs coated with polyacrylic acid (PAA) and poly (allylamine) hydrochloride (PAH)	-	HT-29	-	Alkilany et al. (2009)
	Albumin-coated and fibrinogen-coated GNRs	PTT	MCF-7	-	Hashemi et al. (2019)
	Fetal bovine serum (FBS)-coated and non-fbs-coated GNRs	-	SMCC-7721	-	Ding et al. (2018)
			GES-1		
			4T1		
	GNRs coated with two different densities of SH-PEG	-	-	-	Garcia et al. (2015)
	GNRs conjugated with methylated poly (ethyleneglycol) chains bearing a terminal amine (mPEG-NH_2_)	PTT	KB	-	Huff et al. (2007)
	PSS-, PEG-, mSiO_2_-, dSiO_2_-, TiO_2_-coated GNRs	PTT	HepG2	-	Zhu et al. (2014)
			HT-29		
			U-87MG		
			PC-3		
			MDA-MB-231		
	Arg gly asp (RGD) peptide-functionalized GNRs	PTT	HSC-3	-	Ali et al. (2017c)
	PEG coated GNRs treated with folic acid and loaded with mitoxantrone	Chemotherapy	Hela	-	Nair et al. (2018)
		PTT	C6		
	Short GNRs functionalized with folic acid (FA) and 8-mercaptooctanoic acid (MOA) or 11-mercaptoundecanoic acid (MDA) and loaded with paclitaxel (PCT)	Chemotherapy PTT	MDA-MB-231 MCF-7	-	Papaioannou et al. (2018)
	Human serum albumin/GNRs/doxorubicin/plga	Chemotherapy	CT26	Murine colon cancer	Chuang et al. (2019)
		PTT			
	Hybrid albumin nanoparticles encapsulating small GNRs	PTT	N2a	Glioblastoma N2a tumor-bearing mice	Seo et al. (2019)
	Dual-peptide labeled GNRs	PTT	6606PDA	-	Patino et al. (2015)
	Poly (diallyldimethylammonium chloride)-coated GNRs	-	-	-	Quan et al. (2019)
Reduce the damage to vascular endothelium and systemic toxicity	Multifunctional PEG-b-polypeptide-decorated GNRs	Chemotherapy-PTT	MCF-7	Breast cancer	Hou et al. (2019a)
	Arg-gly-asp (RGD) peptide-functionalized GNRs	PTT	HSC	-	Ali et al. (2017c)
	GNRs linked with rifampicin	PTT	T-U686	Head and neck squamous cell carcinoma (HNSCC)	Ali et al. (2017b)
	Albumin nanoparticles functionalized with folic acid loaded with GNRs and doxorubicin	Chemotherapy-PTT	HeLa	Human cervical cancer	Encinas-Basurto et al. (2018)
	Small GNRs-loaded hybrid albumin nanoparticles	PTT	N2a	Glioblastoma	Seo et al. (2019)
Prolong the blood circulation time	GNRs coated with a zwitterionic stealth peptide	PTT	HepG2	Human liver cancer	Wu et al. (2019a)
	Polysarcosine brush stabilized GNRs	PTT	A549	Human lung cancer	Zhu et al. (2017)
	GNRs modified with folic acid-conjugated block copolymers and Chlorine6(Ce6)	PDT-PTT	MCF-7and A549	-	Choi et al. (2018)
	Ultrasmall GNRs coated with PEG and PLGA	PTT	U87MG	Human glioma	Song et al. (2015)
	Hyaluronic acid-functionalized GNRs	PTT	B16F10.9	Murine melanomas	(Peer and Margalit 2004)
	GNRs-loaded thermosensitive liposome-encapsulated ganoderic acid	Chemotherapy-PTT	MCF-7	Human breast cancer	Zhang et al. (2019)
Enhance passive targeting (EPR effect)	^64^Cu-labeled PEGylated	-	U87MG	Human glioma	Wu et al. (2019a)
	GNRs with different volumes and aspect ratios				
	A dissociable plasmonic vesicle with ultrasmall size (≈60 nm) assembled from small amphiphilic GNRs (≈8 × 2 nm) coated with PEG and PLGA	PTT	U87MG	Human glioma	Wu et al. (2019a)
	A nanoplatform by assembling gold nanorods (GNRs) on the surface of a triangular DNA-origami structure	PTT	4T1	Breast cancer	Wu et al. (2019a)
	Self-assembled DNA origami-GNRs complex	PTT	MCF-7	Breast cancer	Wu et al. (2019a)
	GNRs coated with thiolated PEG	-	-	-	Wu et al. (2019a)
	DOX and GNRs co-loaded polymersomes modified by mPEG-PCL copolymer	PTT-chemotherapy	C26	Mouse colon cancer	Wu et al. (2019a)
	GNRs coated with an enzyme responsive zwitterionic stealth peptide coating consists of a cell penetrating Tat sequence, an MMP-9 cleavable sequence, and a zwitterionic antifouling sequence	PTT	HepG2	Human liver cancer	Wu et al. (2019a)
Enhance active targeting aimed at the tumor-specific receptors	GNRs functionalized with folic acid and 8-mercaptooctanoic acid (MOA) or 11-mercaptoundecanoic acid (MDA) and loaded with paclitaxel	PTT-chemotherapy	MDA-MB-231/MCF-7	Breast adenocarcinoma	Wu et al. (2019a)
	GNRs functionalized with folic acid and loaded with IDO small interfering RNA	PTT-immunotherapy	-	LLC (lewis lung cancer)	Wu et al. (2019a)
	Arg–Gly–Asp (RGD) peptide-functionalized PEGylated	PTT	HSC-3	Human oral squamous cell carcinoma	Wu et al. (2019a)
	GNRs				
	GNRs functionalized with PEG and arg–Gly–Asp (RGD) peptides	PTT	HeLa/MCF-7	Human cervical cancer/Breast cancer	Wu et al. (2019a)
	GNRs combined with 15-polypeptide	PTT	SKOV-3	Ovarian cancer	Singh et al. (2016)
	GNRs linked with anti-cd11b antibodies-decorated NPs	PTT	-	-	Chu et al. (2017)
	Anti-EGFR antibody-conjugated GNRs	PTT	MDA-MB-231	TNBC (triple negative breast cancer)	Zhang et al. (2017c)
	Sialic acid (SA)-imprinted GNRs	PTT	HepG-2	Human hepatoma carcinoma	Yin et al. (2017)
	Hybrid albumin nanoparticles encapsulating small GNRs	PTT	N2a	Glioblastoma	Yin et al. (2017)
	GNRs/DOX/PLGA nanocomplexes coated with human serum albumin (HSA)	PTT-chemotherapy	CT26/MCF7/MCF7-ADR	Mouse colon cancer/Human breast cancer/Multidrug-resistant human breast cancer	Yin et al. (2017)
Enhance active targeting aimed at the tumor-specific pathophysiological conditions	DOX-loaded gold-core silica shell nanorods with salicylic acid and NaHCO_3_ loaded poly (lactic-co-glycolic acid) based microparticles	PTT-chemotherapy	HeLa	Human cervical cancer	Yin et al. (2017)
	A nano-cluster prepared by self-assembling of GNRs conjugated with DOX and amphiphilic poly (curcumin-co-dithiodipropionic acid)-b-biotinylated poly (ethylene glycol)	PTT-chemotherapy	MCF7/MCF7-ADR	Human breast cancer/Multidrug-resistant human breast cancer	Yin et al. (2017)
	Ce6-PEG-GNRs concerning hydrazone bond	PTT-PDT	HeLa	Human cervical carcinoma	Yin et al. (2017)
	A new nanoconstruct composed of GNRs conjugated to carbonic anhydrase IX (CAIX) antibody	PTT	HT29	Human colon adenocarcinoma	Yin et al. (2017)
	GNRs coated with an enzymeresponsive zwitterionic stealth peptide coating consists of a cellpenetrating Tat sequence, an MMP-9 cleavable sequence, and a zwitterionic antifouling sequence	PTT	HepG2	Human liver cancer	Yin et al. (2017)
	A protein-free collagen nanosweeper, triphenylphosphonium bromide (TPP) coated and S-nitrosothiols loaded mini-sized Au@silica nanorod	PTT	HeLa/4T-1/MCF-7	Human cervical cancer/Breast cancer	Yin et al. (2017)
	GNRs functionalized with hyaluronic acid (HA) bearing pendant hydrazide and thiol groups via Au-S bonds and conjugated with 5-aminolevulinic acid (ALA), Cy7.5 and anti-HER2 antibody	PTT-PDT	MCF-7	Breast cancer	Yin et al. (2017)
	Disulfiram- GNRs integrate	PTT-chemotherapy	MCF-7	Breast cancer	Yin et al. (2017)
Enhance cell-mediated targeting	Human CIK cells loaded with silica-coated GNRs	PTT-immunotherapy	MGC803	Gastric cancer	Yang et al. (2016b)
	GNRs-loaded platelets	PTT	CAL27	HNSCC (head and neck squamous cell carcinoma)	Yang et al. (2016b)
	Macrophages loaded with GNRs and DOX-LPs	PTT-chemotherapy	4T1	Breast cancer	Yang et al. (2016b)
	Macrophage-loaded Anionic-GNRs	PTT	4T1	Breast cancer	Yang et al. (2016b)
	Human induced pluripotent stem cells loaded with GNRs @SiO2@CXCR4 nanoparticles	PTT	MGC803	Gastric cancer	Yang et al. (2016b)
	Human induced pluripotent stem cells loaded with GNRs @SiO2@CXCR4 nanoparticles which were pre-treated with mitomycinC (MMC)	PTT	MGC803	Gastric cancer	Yang et al. (2016b)
Enhance homologous targeting (cancer cell membrane-mediated targeting)	Cancer cell membrane-coated GNRs	PTT-radiotherapy	KB	Human oral squamous cancer	Yang et al. (2016b)
	Cancer cell membrane loaded with a biodegradable nanogel crosslinked by cisplatin (CDDP) and functionalized with GNRs and DOX	PTT-chemotherapy	4T1	Breast cancer	Yang et al. (2016b)
Improve the tumor killing effect	Chitosan-conjugated, pluronic-based nanocarriers with GNRs	PTT	SCC7	Squamous carcinoma	Choi et al. (2011)
	GNRs and doxorubicin co-loaded polymersomes	PTT and chemotherapy	C26	Colon cancer	Liao et al. (2015)
	GNRs/chlorin e6(Ce6) loaded stem cell system	PTT and PDT	CT26	Colon cancer	Chuang et al. (2020)
	Zinc phthalocyanine loaded GNRs	PTT and PDT	Hela	Cervical cancer	Tham et al. (2016)
			MCF-7	Breast cancer	
	GNRs、folic acid、Ido small interfering RNA nanocomplex	PTT and immunotherapy	LLC	Lung cancer	Zhang et al. (2020)
	Pyrene-aspirin loaded GNRs	PTT and anti-inflammatory therapy	4T1	Breast cancer	Dong et al. (2018)
Construct a pH/NIR triggered drug release system	A novel pH sensitive targeted polysaccharide-GNRs conjugate	Photothermal-chemotherapy	MCF-7	Breast cancer	Liu et al. (2015)
	GNRs/mSiO_2_ combined with PH responsive polyhistidine	Photothermal-chemotherapy	SW620	Human colon cancer	Jiang et al. (2020)
	Hyaluronic acid-functionalized GNRs	Photothermal-chemotherapy	MCF-7	Breast cancer	Xu et al. (2017)
	GNRs/hydrogel core/shell nanospheres	Photothermal-chemotherapy	PC-3	Human prostate cancer	Jin et al. (2015)
Construct a redox/pH/NIR triggered drug release system	GNRs-based complexes containing hydrazine and disulfide bonds	Photothermal-chemotherapy	MCF-7	Breast cancer	Hou et al. (2019a)
	Disulfiram-GNRs	Photothermal-chemotherapy	MCF-7	Breast cancer	Xu et al. (2020)
	PH/Redox responsive core cross-linked nanoparticles from thiolated carboxymethyl chitosan	Photothermal-chemotherapy	HeLa	Human cervical cancer	Gao et al. (2014)
	GNRs and docetaxel based nanoparticles coated with ultra-thin MnO_2_ nano-film	Photothermal-chemotherapy	MCF-7	Breast cancer	Wang et al. (2017)

### Cellular Uptake

**FIGURE 2:**
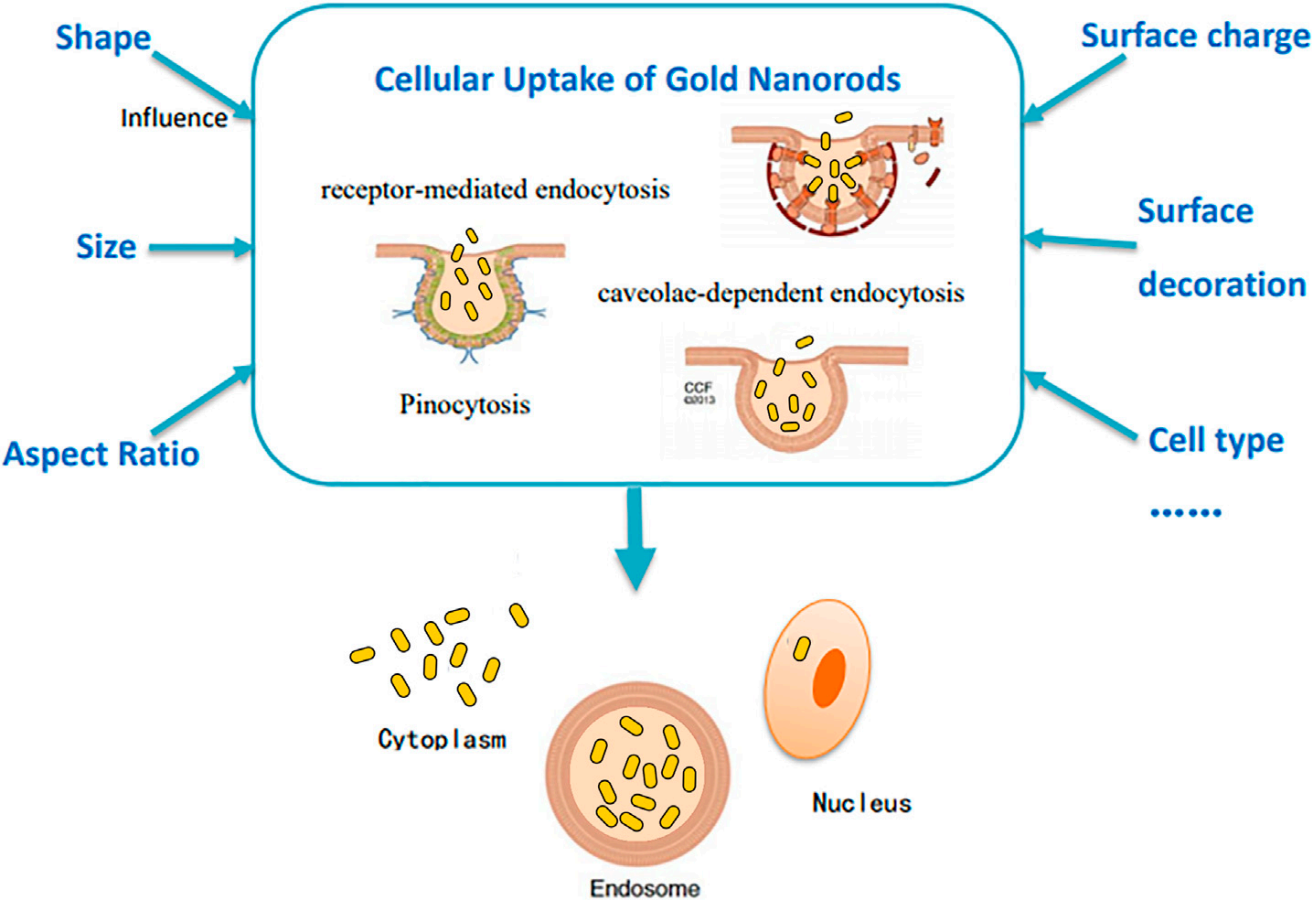
Illustration of several factors affecting the cellular uptake of GNRs.

Effective cellular uptake dependent on various physicochemical and biological parameters (Yang H. et al., 2016) is the basis of biological applications of GNRs. The cellular uptake of nanoparticles occurs after their interaction with cell membranes (Sun et al., 2018). Cells internalize GNRs through different mechanisms of endocytosis, including receptor-mediated endocytosis (RME; Chithrani et al., 2006), clathrin-mediated pinocytosis (Favi et al., 2015), caveolae and dynamin-dependent micropinocytosis (Saha et al., 2013), etc., which are influenced by cell types and characteristics of nanorods (Zhang et al., 2015). After entry into cells, the rod shape is maintained (Favi et al., 2015; Gao et al., 2016; Mahmoud NN. et al., 2019). Most nanorods scatter or aggregate in the cytoplasm or can be internalized in membrane-bound vesicles (Chithrani et al., 2006; Favi et al., 2015), stay in the endosome (Yang H. et al., 2016), and move through an endolysosomal pathway for degradation (Jiang et al., 2008; Chithrani 2010). A few GNRs clustered in the perinuclear zone and were allowed to enter the nucleus (Abdelrasoul et al., 2016). The following points can affect the cellular uptake of GNRs in different ways (Figure 2).

#### Shape and Size

The shape and size of nanorods cause a significant impact on cellular uptake. Untargeted gold nanospheres can enter the cell more effectively than untargeted rod-shaped gold nanoparticles (Chithrani et al., 2006; Yang H. et al., 2016), whereas short nanorods modified with targeting ligands enter cells more effectively than their spherical counterparts (Favi et al., 2015; Yang H. et al., 2016). For instance, phospholipid-polyethylene glycol (PEG)-GNRs designed by Mahmoud et al. displayed higher cellular uptake efficiency (Mahmoud N. N. et al., 2019). In contrast, Lee et al. found that the uptake of chitosan-capped GNRs was lower than chitosan-coated gold nanospheres (Lee et al., 2019). In addition, it is suggested that a size range of 20–50 nm is favorable for cellular uptake (Jiang et al., 2008; Alkilany and Murphy 2010; Chithrani 2010; Bandyopadhyay et al., 2018), with the maximum uptake falls on 50 nm nanoparticles (Chithrani et al., 2006; Jiang et al., 2008). It is speculated that differences in the degree of nanoparticles entering cells are attributed to the competition between the membrane wrapping and receptor diffusion kinetics (Chithrani et al., 2006; Lee et al., 2019). Therefore, both extremely small and large nanoparticles would lead to inefficient uptake (Jiang et al., 2008), because small nanoparticles often lack the binding capability of ligands with receptors, and the slow receptor diffusion of large nanoparticles leads to short wrapping time and low efficiency of cellular uptake (Chithrani et al., 2006; Jiang et al., 2008).

**FIGURE 3:**
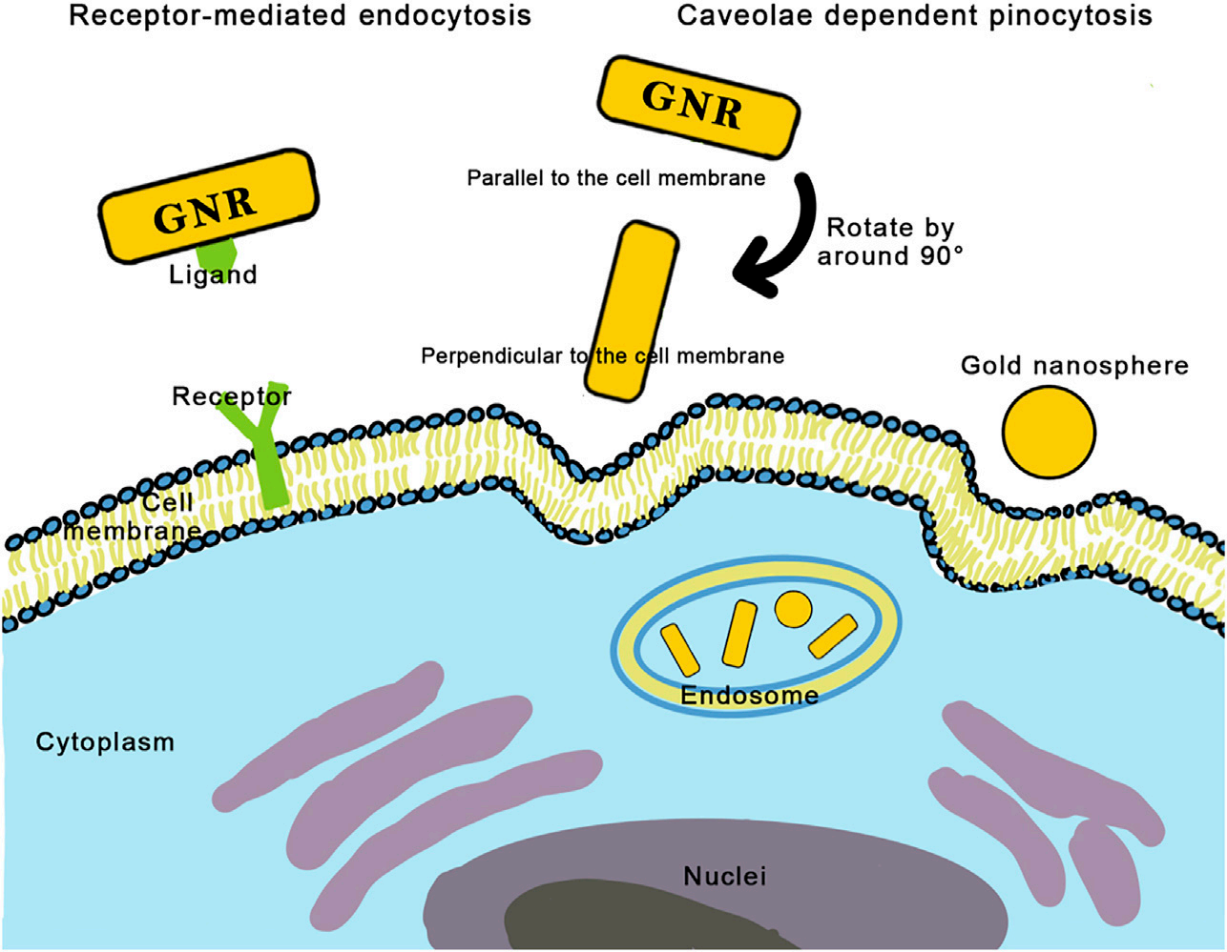
Two mechanisms of cellular uptake of gold nanorods: receptor-mediated endocytosis and caveolae dependent pinocytosis. Long nanorods pre-align to the cell membrane almost parallelly, then rotate by around 90° to enter the cell, while short nanorods can directly be entrapped by cells without rotation.

As the AR increases, the cellular uptake of nanorods decreases (Chithrani et al., 2006; Chithrani 2010; Qiu et al., 2010; Yang H. et al., 2016). Yang et al. believed that DNA-coated gold nanoparticles with different ARs enter the endothelial cells via the same caveolae-mediated pathway and found that ARs influenced the orientation of GNRs. Long DNA-coated nanorods prealign to the cell membrane almost parallelly and then rotate by about 90° to enter the cell, whereas short nanorods can directly be entrapped by cells without rotation (Figure 3; Yang H. et al., 2016). Qiu et al. also found that long nanorods tended to form larger aggregates with loose structures, requiring more energy consumption for endocytosis (Qiu et al., 2010).

#### Surface Charge and Decoration

Surface chemistry, determined by factors such as surface charge, hydrophilicity, and surface functionalization, shows vital importance in the interaction between GNRs and cells.

GNRs bearing cationic structures bind to the cell membrane and are taken up by cells more efficiently than GNRs bearing anionic structures due to the electrostatic interaction between the positively charged surface and the negatively charged cell membrane (Chithrani et al., 2006; Alkilany et al., 2012; Kah et al., 2014; Sun et al., 2018; Zarska et al., 2018). Materials with hydrophilicity display better interaction with cell membranes to some degree (Kah et al., 2014). However, a preferential uptake for the negatively charged nanoparticles was reported by Patil et al. and Mahmoud et al. (Patil et al., 2007; Mahmoud NN. et al., 2019), which could be explained by the fact that proteins absorbed from biological media form a “protein corona,” altering the surface chemistry and size of the associated nanoparticles, thus enhancing or retarding their cellular uptake (Alkilany et al., 2009; Alkilany and Murphy 2010; Kah et al., 2014; Mahmoud NN. et al., 2019). The properties of nanoparticles, as well as the environment, affect the proteins adsorbed on the surface of nanoparticles (Kah et al., 2014; Favi et al., 2015; Hashemi et al., 2019), and both the types and amount of absorbed proteins influence the cellular uptake mechanism (Ding et al., 2018; Sun et al., 2018). Proteins may induce nanoparticles to enter the cells via the protein-RME (Alkilany et al., 2012; Abdelrasoul et al., 2016).

Traditionally synthesized GNRs were coated with CTAB, and displacing CTAB with PEG chains greatly reduced the uptake (Huff et al., 2007; Qiu et al., 2010; Alkilany et al., 2012; Garcia et al., 2015; Sun et al., 2018). Phospholipid-coated GNRs could enhance the uptake of nanorods due to their biochemical affinity to the cell membranes (Mahmoud N. N. et al., 2019). Inorganic-coated GNRs also demonstrated high cellular uptake (Zhu et al., 2014). GNRs decorated with targeting ligands endow the quick contact with receptors overexpressed on the membrane in some cancerous cells (Gao et al., 2016). Therefore, polypeptides targeting integrins (Ali et al., 2017c), folic acid (FA; Gao et al., 2016; Nair et al., 2018; Papaioannou et al., 2018), human serum albumin (has; Chuang et al., 2019), and hybrid albumin (Seo et al., 2019) were utilized to improve the uptake efficiency. Dual-peptide (Glu-Pro-Pro-Thr + myristoylated polyarginine peptide) labeled GNRs combined the effects of targeting and electrostatic and hydrophobic interaction to show significantly higher cellular uptake (Patino et al., 2015). In addition, Quan et al. (2019) believed that a higher density of ligands attracted more GNRs to the cell membrane.

Because multiple parameters, such as cell types (Zhu et al., 2014; Zhang et al., 2015; Mahmoud N. N. et al., 2019), medium components (Kah et al., 2014), incubation time (Quan et al., 2019), and concentration (Chithrani et al., 2006; Garcia et al., 2015), affect the cellular uptake, there are contradictions when studying its influencing factors, and the different conditions applied in separated experiments made it impossible to directly compare the uptake of different GNRs. Major cellular uptake experiments are conducted *in vitro*, which distinguishes it from the *in vivo* environment (Dobrovolskaia and McNeil 2007). Meanwhile, the study of cellular uptake mechanism and intracellular trafficking of GNRs are critical as they provide useful information (Zhang et al., 2015), which are still poorly understood. Therefore, more attention should be paid to *in vivo* experiments and mechanisms in further research to prepare optimized GNRs for treatments.

### Blood Circulation

GNRs hold great prospects in the medical domain for their therapeutic effect on cancer, but their application *in vivo* also faces great challenges. One of the critical problems is the short blood circulation time. The long blood circulation time can ensure that the nanoparticles accumulate passively or actively in the tumor site. Thus, it is necessary to find a new strategy to prolong the blood circulation time of nanoparticles, which will greatly improve its therapeutic effect. Because GNRs are designed for intravenous injection and particularly relevant to the blood system, the cells of the blood system will be one of the first biological systems to be exposed to the injected nanomedicine, and blood compatibility is essential for GNRs. The full development of nanotechnology in pharmaceutical products still requires efforts to transform it from a laboratory environment to a clinical application. Because toxicity accounts for 20% of all drug failures in clinical trials, it must be considered as potential factors for the failure of nanomaterials (Kola and Landis 2004), and the local therapeutic nanocomposites with the advantage of low systemic toxicity exhibit a more promising prospect (Jiang et al., 2019). Therefore, recent research on reducing the systemic toxicity of GNRs and prolonging their blood circulation time, enhancing the therapeutic effect, is introduced.

#### Reduce Toxicity to Endothelial Reticular Cells

GNRs are promising agents in biomedical applications, such as sensing, imaging, drug delivery, and cancer therapy, but their biosecurity remains to be an unsolved and controversial problem to some extent. Qiu et al. looked into the effect of AR and surface coating on the toxicity of GNRs. Their data showed that the cytotoxicity was independent of shape but related to the surface coating (Qiu et al., 2010), which was also confirmed by Jiang et al. (2020). The methods of reducing the concentration of CTAB, which mainly caused the cytotoxicity of GNRs, have been mentioned above, chiefly by cleaning repeatedly or utilizing nontoxic surfactants in the industrial production process. This study focused on the improved surface functionalization of GNRs to reduce toxicity. For example, PEG-modified gold nanoparticles can reduce the damage to the vascular endothelium (Hou et al., 2019a). Ali et al. cleaned GNRs twice to remove CTAB and did surface functionalization using PEG and Arg-Gly-Asp (RGD) peptide to eliminate the toxicity of GNRs (Ali et al., 2017c). They also linked rifampicin (RF) to GNRs, which proved to lower toxic effects (Ali et al., 2017b). Seo et al. directly replaced CTAB with thio-bovine serum albumin (BSA-SH), synthesizing a novel material to reduce toxicity, and it is suitable for glomerular excretion (Seo et al., 2019). Positively charged substances can nonspecifically bind to most cells in circulation, leading to low drug accumulation in tumor sites and significant adverse effects on normal tissues (Han et al., 2015). A negatively charged biomaterial in the body fluids, serum albumin, exhibits superior biocompatibility and biodegradability. Studies have shown that serum albumin-covered GNRs can dramatically circumvent the cytotoxic effects induced by CTAB (Encinas-Basurto et al., 2018). To sum up, the surface functionalization of GNRs has a great impact on reducing the toxicity of residual CTAB. Although many surface modifiers have been found, further efforts are needed to develop a more simple, inexpensive, and green one, worthier for clinical use.

#### Prolong the Time of Blood Circulation

Compared to indocyanine green and other photothermal materials, the removal rate of GNRs *in vivo* is relatively slow (Ishizawa et al., 2009). Long blood circulation time, which ensures that nanoparticles passively or actively accumulate in the tumor sites, is essential for effective drug delivery and anticancer therapy (Davis et al., 2008; Zhang Z. et al., 2014). To achieve long-lasting blood circulation, “stealth” coatings, such as hydrophilic substances and even cell membranes, are introduced to stabilize nanomaterials and increase their solubility, thus prolonging blood retention and reducing unnecessary uptake in the reticuloendothelial system (RES). However, these stealth coatings may significantly reduce the contact between nanomaterials and cells in the tumor region, thereby reducing endocytosis. Achieving long blood circulation time of nanomaterials while maintaining enhanced cancer cellular uptake remains a great challenge. Only by taking both the two factors into account can a good therapeutic effect be achieved.

One feasible measure is to attach tumor-targeting groups together with antifouling coatings, which inhibit nonspecific binding during the cycling of nanomaterials. When the complex reaches the tumor sites, the protective layer can be automatically removed to expose the internal targeting groups, thus promoting cellular uptake (Olson et al., 2010; Wang S. et al., 2016; Sun et al., 2016; Adamiak et al., 2017; Han et al., 2017; Piao et al., 2018). This coating is similar to the stealth coating. When it reaches the tumor site’s microenvironment, the properties of the coating are transformed to increase blood circulation while increasing cell endocytosis in the tumor region (Zhu et al., 2013; Ohta et al., 2016; Parak 2016). Similarly, Wu et al. developed a zwitterionic stealth peptide coating that can respond to the tumor areas overexpressed matrix metalloproteinase-9 (MMP-9). The peptide consists of a Tat sequence, an MMP-9 cleavable sequence, and a zwitterionic antifouling sequence. It was bound to GNRs by ligand exchange to achieve long blood circulation time and high tumor accumulation. In detail, a highly cationic peptide sequence (GRKKRRQRRPQ) extracted from the Tat protein was used to construct the inner layer of the membrane. The Tat peptide can effectively penetrate cells and thus mediate the delivery of protein, nucleic acid, and nanoparticles. An MMP-9-sensitive peptide sequence was used as a bridge linking cell-penetrating peptide and antifouling peptide sequences to form a tumor-responsive peptide coating. To stabilize the nanomaterials, a zwitterionic peptide sequence was introduced on top of the response coating to act its good antifouling properties (Wu L. et al., 2019). Meanwhile, it has been reported that hydroxyethyl chitosan-coated nanoparticles are formed by cationic and anionic polymers and exhibit pH-sensitive surface charge reversal behavior, which can result in prolonged blood circulation time and fewer side effects (Peer and Margalit 2004). This is similar to the principle of amphoteric peptides mentioned above. Additionally, PEG-modified nanomaterials showed prolonged retention in circulation and increased solubility, and unnecessary uptake in the RES can be effectively reduced. Its effect of prolonging blood circulation is the same as that of the amphoteric peptide (Choi et al., 2018).

Reducing the material size is also a meaningful way to prolong blood circulation. Song et al. previously developed PEG and poly (lactic-co-glycolic acid) (PLGA) mixed brush-coated amphiphilic GNRs and further assembled them into biodegradable plasmonic vesicles for thermosensitive applications. The relatively huge vesicles (>200 nm), however, limit the local distribution of drugs, because the intravenous injection can lead to the rapid accumulation of drugs in the RES or liver and spleen. Individual GNRs with a width more than 8 nm and a length of about 40 nm are not readily excreted from the body even if the vesicles can degrade over time (Zhang Z. et al., 2014). The ideal plasma assembly with the size of sub-100 nm should consist of smaller GNRs and biocompatible materials. To overcome the above shortcomings, a novel plasmonic vesicle with minimal size (≈60 nm), which is biocompatible and dissociable, assembled by amphiphilic GNRs decorated with PEG and PLGA was proposed to prolong blood circulation and gain effective accumulation in tumor regions based on the enhanced permeability and retention (EPR) effects. AuNR@PEG/PLGA vesicles were degraded into small GNRs (AuNR@PEG) that are hydrophilic after PLGA hydrolysis, which were stable under physiological conditions and can be easily removed from the body (Song et al., 2015).

Increasing the blood circulation time of the material can be realized not only by adding stealth coating and improving the size of the material but also by changing the hydrophilicity of the material and increasing the water solubility. Xu et al. synthesized hyaluronic acid (HA)-functionalized GNRs, which show a relatively long blood circulation time. In addition, 24 h after administration, GNRs-HA-FA-doxorubicin (DOX) showed a higher retention rate in circulation than the previously reported GNRs of PEG, SiO2, and CS modifications (Chen et al., 2013; Black et al., 2014; Zhang Z. et al., 2014). The hydrophilicity and immunosuppressive property of HA grant GNRs-HA-FA-DOX excellent blood circulation time and retention rate (Peer and Margalit 2004). Zhang et al. wrapped self-assembled GNRs and *Ganoderma* acid A (Ga.A) into thermosensitive liposomes (LTSL). They used LTSL to coat Ga. A that effectively changed the polarity of Ga. A and showed good water solubility, exhibiting prolonged circulation time (Zhang et al., 2019).

To sum up, increasing the size of the material using stealth coating or other surface functionalization can reduce the interaction between the material and the blood system or other tissue systems and further prolong the blood circulation time of GNRs materials. Many studies have taken the two aspects into account: prolonged blood circulation and increased endocytosis of tumor cells. However, further exploration is needed in terms of specific mechanisms. The PTT of GNRs is a coherent process. More systematic studies are needed to explore other effects of the surface functionalization mentioned above, and comprehensive consideration is needed to enhance the PTT effect of GNRs.

### Tumor Tissue Accumulation

**FIGURE 4:**
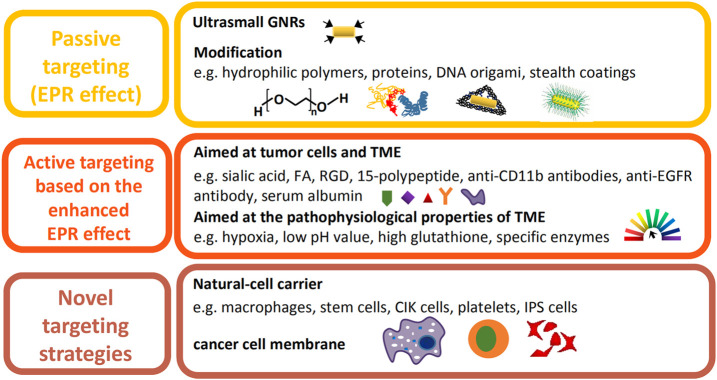
Illustration of recent improvements on the tumor accumulation of GNRs.

The high concentration of drugs in the tumor site plays a key role in cancer therapy (Park et al., 2019), while unspecific targeting leads to low bioavailability and systemic toxicity. In various studies, based on the EPR effect, GNRs are modified into different sizes and shapes, or surface-functionalized with specific targeting agents, to increase the accumulation in tumor tissue. Besides, novel targeted delivery systems, such as cell-mediated targeting and homologous targeting, have drawn significant attention due to the capacity to overcome the disadvantages of traditional strategies. Therefore, recent studies that improve the tumor accumulation of GNRs are summarized as follows (Figure 4).

#### Passive Targeting

**FIGURE 5:**
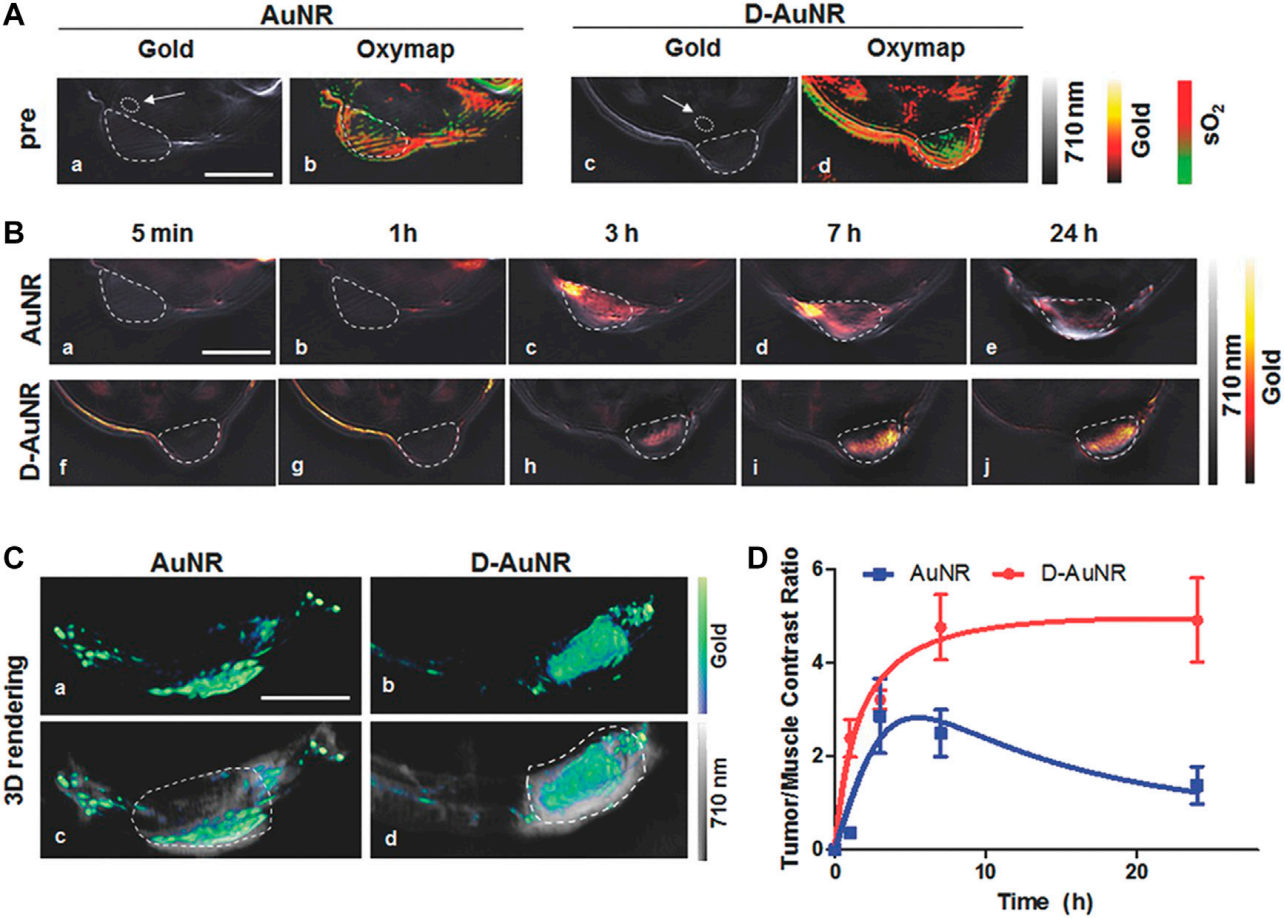
Optoacoustic evaluation of DNA-Nanostructure-Gold-Nanorod Hybrids which achieve better accumulation in tumor sites than pure GNRs. **(A)** The gold distribution (hot scale, a,c) and the corresponding oxygen-saturation maps (green to red scale, b,d) before intravenous injection of GNRs and GNRs with DNA nanostructures (D-GNRs) in 4T1-tumor-bearing mice. **(B)** GNRs and D-GNRs distribution (hot scale) at several time points including 5 min (a,f), 1 h (b,g), 3 h (c,h), 7 h (d,i), 24 h (e,j) after intravenous injection in 4T1-tumor-bearing mice (dashed outlined) overlayed on an optoacoustic image acquired at a single illumination wavelength (710 nm, gray scale). Scale bar = 5 mm. **(C)** 3D rendering the optoacoustic images in the cancerous regions on 4T1-tumor-bearing mice 24 h postinjection of the GNRs (a, hot scale) and D-GNRs (b, hot scale), overlayed on single wavelength images (c,d, 710 nm, gray scale). Scale bar = 5 mm. **(D)** Contrast ratio between the tumor and the region of back muscles extracted from the images for GNRs (blue) and D-GNRs (red). A section of the back muscle (indicated by the white arrows) is outlined in the initial single wavelength image (Du et al., 2016b).

The leaky blood vessels and poor lymphatic drainage within solid tumors allow for the accumulation of nanoparticles of specific sizes and shapes in the tumor region, which is known as the EPR effect. However, the intensity of the EPR effect varies with the type, location, host, and stage of a certain tumor, affecting drug delivery efficiency and therapeutic outcome (Bertrand et al., 2014). Additionally, the physicochemical properties of a nanocarrier, such as size, shape, and hydrophilia, result in the heterogeneity of the EPR effect (Park et al., 2019). GNRs of smaller volume and higher AR, especially when packaged into sub-100 nm-sized plasmonic assemblies with biocompatible materials, contribute to the enhanced accumulation at the tumor site and the rapid excretion from the body after therapy (Song et al., 2015; Tong et al., 2016). A novel nanocarrier, DNA-origami, especially triangle-shaped origami, loaded with GNRs, facilitated the accumulation of drugs in the tumor tissue (Figure 5; Du et al., 2016a; Jiang et al., 2015). Moreover, hydrophilic polymers (Alkilany et al., 2012; Liao et al., 2015), proteins, and stealth coatings (Wu L. et al., 2019) are usually conjugated to the surface of GNRs, improving the EPR effect by prolonging systemic circulation. Due to the unsatisfactory therapeutic efficacy of EPR-dependent nanomedicines mostly caused by the heterogeneity of the EPR effect, effective measures should be taken to enhance it.

#### Active Targeting Based on the Enhanced EPR Effect

GNRs nanoplatforms functionalized with antibodies (Chu et al., 2017), peptides (Singh et al., 2016), proteoglycan, vitamins (Papaioannou et al., 2018; Zhang et al., 2020), and aptamers realize selective targeting via binding to the specific receptors of tumor cells or the TME. To overcome the disadvantages of natural antibodies, such as poor stability, comparably complicated preparation process, and low affinity toward nonimmunogenic targets (Ye and Mosbach 2008), molecular imprinting technology was exploited to construct sialic acid (SA)-imprinted GNRs, which exhibited high affinity to cancer cells overexpressed SA (Yin et al., 2017). Additionally, increasingly more studies have focused on the potential molecular targets in TME. Serum albumin, capable of binding to gp60 receptors expressed on tumor vascular endothelial cells, was added to GNRs-based nanomedicines to achieve long blood circulation time and more intracellular accumulation of chemotherapeutic agents (Chuang et al., 2019; Seo et al., 2019). According to Huang et al., the combination with active targeting ligands did not significantly enhance the total tumor uptake of gold nanoparticles, simply affecting their distribution in tumor cells and the TME, which implied that the above active targeting agents are probably not the best choices (Huang et al., 2010).

**FIGURE 6:**
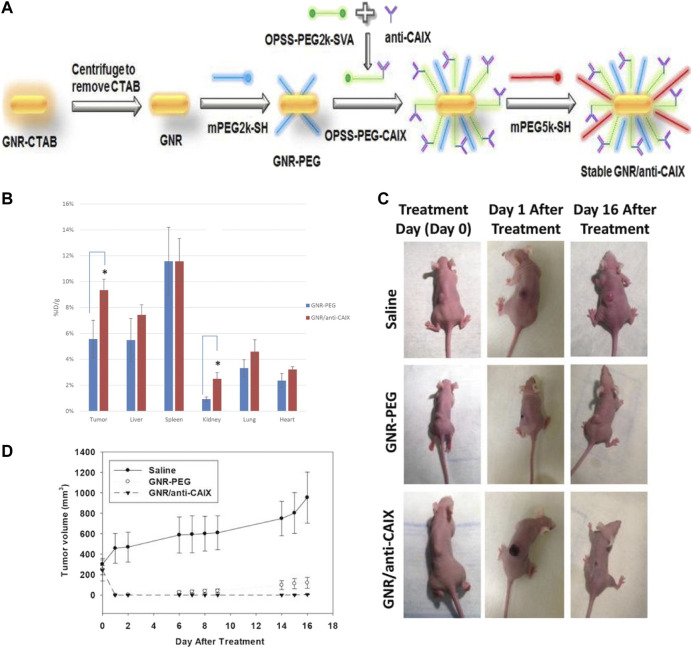
The preparation and properties of hypoxia-targeted GNRs. **(A)** The preparation of hypoxia-targeted GNRs. The conjugation of GNRs with anti-CAIX antibody via bi-functional crosslinker. **(B)** Comparison of gold content in tissues by Inductively Coupled Plasma-Mass Spectrometry (ICP-MS) 24 h after intravenous administration of targeted (GNRs/anti-CAIX) and untargeted (GNRs-PEG) GNRs in HT29-tumor-bearing mice (*n* = 4 for both groups). The uptake of GNRs/anti-CAIX was significantly higher than GNRs-PEG in xenograft tumor (*p < 0.05). The biodistribution in other organs was similar for both groups except higher uptake in the kidney for GNRs/anti-CAIX. **(C)** Photothermal ablation via near infrared irradiation of HT29 tumors 24 h after tail-vein injection of saline (n = 3), GNRs-PEG (*n* = 5, OD = 20) or GNRs/anti-CAIX (*n* = 7, OD = 20). Images of representative mice in each group prior to treatment, one day after treatment, and 16 days after treatment. Tumor volume plotted over time for all three groups. **(D)** No tumor regression in the saline-treated group; regression but recurrence of tumor in the GNRs-PEG treated group; and complete tumor regression in the GNRs/anti-CAIX treated group (Chen et al., 2018a). (Hypoxia-targeted gold nanorods for cancer photothermal therapy, https://creativecommons.org/licenses/by/3.0/).

Many researchers have recognized the pathophysiological properties of TME, such as hypoxia, low pH value (Moreira et al., 2017; Wang et al., 2018; Zhang C. et al., 2017), high concentration of GSH, and specific enzymes, as ideal targets toward cancer therapy. Carbonic anhydrase IX (CAIX), a transmembrane protein highly expressed in hypoxic zones, is critically involved in the cellular migration and metastasization of cancer cells. According to Chen et al., GNRs decorated with anti-CAIX antibodies exhibited preferential targeting to hypoxic tumor cells harboring cell-surface CAIX protein, which facilitated the selective ablation of these cells via PTT (Figure 6; Chen et al., 2018b). Interestingly, Fulvio et al. found that GNRs showed higher accumulation rates when conjugated with sulfonamides that act as inhibitors toward CAIX than conjugated with anti-CAIX antibodies, inducing the sensitization to subsequent optical ablation (Ratto et al., 2014). However, nanoparticles designed based on hypoxia of TME have been limited in clinical use, as the extent of hypoxia varies dramatically between tumors, leading to unpredictable therapeutic outcomes, for which the artificial induction of hypoxic stress or multitargeting strategies might be promising approaches (Park et al., 2019). Nanocarriers targeting TME-specific enzymes have been another research focus currently. Wu et al. developed a GNRs-loaded enzyme-responsive multifunctional peptide coating, the middle layer of which was sensitive to MMP-9, which is overexpressed in TME, inducing the degradation of the outer layer and penetration into tumor cells (Wu L. et al., 2019). Liu et al. constructed a triphenylphosphonium bromide (TPP)-coated mini-sized Au@silica nanorod, loading S-nitrosothiols, which induced the release of NO upon NIR laser irradiation, activating MMP-1 and -2 in the TME and subsequently eliciting collagen depletion and deeper penetration of nanomedicine into the tumor site (Liu et al., 2020). Moreover, multistimuli-responsive theragnostic nanoplatforms could also greatly improve the targeting efficiency (Xu et al., 2019).

#### Novel Targeting Strategies

Traditional nanodrug delivery systems based on the EPR effect and the binding of ligands and receptors have shown evident shortcomings in clinical use, such as low targeting efficiency, poor stability, and potential immunotoxicity. Recently, studies on GNRs-loaded nanoparticles carried by several natural cells such as macrophages (Walsh et al., 2017a; Borri et al., 2018), stem cells, cytokine-induced killer cells (CIK; Yang Y. et al., 2016), and platelets (Rao et al., 2018), which exhibit their deep penetration into the tumors as well as low immunogenicity and toxicity, have caught researchers’ attention. Macrophages are considered to be ideal carriers of nanomedicines due to their ability of natural phagocytosis, migration through the blood barrier, and targeting tumors. Nguyen et al. constructed a macrophage-based nanoplatform loaded with small-sized GNRs and DOX-containing nanoliposomes, which showed enhanced tumor coverage and deeper penetration into the 3D cancer spheroid model (Nguyen et al., 2020). According to An et al., the designed macrophage-loaded anionic GNRs facilitated cellular uptake and exhibited a macrophage-involved tendency to penetrate deeper into the hypoxic regions of tumors (An et al., 2019). Thus, the macrophage-mediated drug delivery system might overcome the therapeutic difficulties in hypoxic regions of tumors, which exhibit poor susceptibility to anticancer drugs, radiation, and free radicals. Similarly, stem cells have shown their potential in cell-mediated GNRs-loaded drug delivery as a result of the intrinsic characteristics of targeting tumors. Liu et al. fabricated a GNRs@SiO_2_@CXCR4 nanoplatform and loaded it into human-induced pluripotent stem cells (iPS). The enhanced migration from the injection site to the tumor site was observed in MGC803 tumor-bearing mice (Liu et al., 2016). To reduce the iPS risk of forming teratoma, Liu et al. further studied the inhibitory effect of mitomycin C on iPS. Mitomycin C-treated iPS in organs died after 7 days of PTT, exhibiting enough safety and simultaneously remarkable therapeutic efficacy (Yang et al., 2017).

Additionally, cancer cell membrane-camouflaged GNRs-loaded nanoparticles possess the capacity of immune escape and homologous targeting, mainly owing to the specific membrane proteins (Hanahan and Weinberg 2011), overcoming the disadvantages of traditional targeting agents, such as short blood circulation time, nonspecific binding, and immune clearance (Zhen et al., 2019). However, clinical translation of cancer cell membrane-based nanoparticles is not easy due to some unsolved problems. First, numerous proteins are expressed on tumor cell membranes, among which only a few play key roles in homologous targeting, whereas others would induce immune responses and adverse effects. Therefore, how to remove irrelated proteins is still a valuable question that deserves to be solved. Second, the relatively complex preparation process and the low yield of cancer cell membranes limit large-scale production. One group considered that the development of microfluidic technologies could be used to tackle manufacturing issues and promote clinical translation (Bose et al., 2018). Third, nanoparticles coated with cancer cell membrane cannot deeply penetrate the tumors, hindered by the dense extracellular matrix and rising interstitial fluid pressure in the tumor region. The surface functionalization of active targeting agents such as specific peptides would help enhance the permeability ability of nanoparticles. Last, the long-term biological effects of cancer cell membrane-camouflaged nanomaterials on healthy tissues should be further investigated (Zhen et al., 2019).

## Tumor-Killing Effect

### Photothermal Therapy

Hyperthermia is a clinical method used to kill tumor cells. Treatment at 50°C for 4–6 min or 42–45°C for 15–60 min can effectively kill tumor cells (Sapareto and Dewey 1984; Habash et al., 2006). However, traditional hyperthermia uses microwaves, ultrasound, and magnetic fields as heat sources to nonselectively irradiate the tumor site, which cannot guarantee tumor-specific treatment. While killing the tumor, it also damages the surrounding normal tissues (Vankayala and Hwang 2018). The discovery of photothermal agents in recent years has greatly increased the specificity of hyperthermia. A photothermal agent is a substance that can be injected into the body and accumulate at the tumor site through targeted modification and generate heat energy under external stimulation to increase the local temperature of the tumor. Among various photothermal agents, GNRs show a good photothermal conversion effect, owing to the SPR effect, and act as a better therapeutic material with their ability to absorb NIR, which can effectively penetrate healthy tissues (Lal et al., 2008; Bagley et al., 2013). The main advantages of PTT include less invasion than surgical treatment, high penetrability into deep tumor tissues due to the characteristics of NIR light, low toxicity, and high targeting ability, owing to proper surface functionalization of the contrast agent and the spatiotemporal control of treatment achieved through light irradiation (Vankayala and Hwang 2018). The main mechanism of PTT is that local high temperature induces a series of changes in cells, which leads to apoptosis or necrosis of tumor cells. Normal cells can be in a state of heat stress under high temperature, during which the expression of heat shock protein (HSP) increases, which reduces the damage caused by protein denaturation and inhibits the activation of apoptosis-related pathways simultaneously, so that cells reach an adaptive state (Beere 2004; Lanneau et al., 2008). However, when the temperature is too high and exceeds the cell adaptation range, the expression of HSP decreases. At this time, tumor necrosis factor (TNF)-related apoptosis-inducing ligand, caspase, Fas ligand, and TNF-α are overexpressed, causing the cells to undergo apoptosis. Moustafaet et al. studied different pathways that mediate apoptosis through quantitative proteomics analysis and discovered the important role of cytochrome C and p53-related pathways (Ali et al., 2017a). Song et al. utilized patch-clamp technology and discovered that local high temperature could open the TRPV1 ion channel on the cell membrane, induce excessive calcium influx, and activate the protease caspase-9 to cause cell apoptosis (Song et al., 2020). PTT can not only effectively kill local tumor cells. Wu et al. also found that the introduction of GNRs and NIR can inhibit the collective migration of tumor cells by changing the actin filaments and cell-to-cell connections, thus improving the prognosis of patients (Wu et al., 2018). Nabil et al. fabricated an improved mathematical model to investigate the delivery and hyperthermia effect of nanoparticles in cancer treatment, where they identified that perfusion and diffusion are two factors mediating the distribution of the particles and heat. Their study provided insights for a better underlying mechanism of hyperthermia (Nabil et al., 2015).

Gold nanoparticles of different shapes (GNRs, gold nanoshells, gold nanocages, gold nanospheres, etc.) possess different light-to-heat conversion efficiency, among which anisotropic GNRs display the highest efficiency (Von Maltzahn et al., 2009). To investigate the association between the size of GNRs and their photothermal conversion efficiency, Mackey et al. found that 28 × 8 nm GNRs have the best photothermal conversion efficiency through theoretical calculations and experiments. In practical applications, GNRs are often modified to form a multifunctional nanocomposite. Won Il Choi et al. loaded GNRs into nanocarriers to improve their performance. After injection into mice, they were exposed to laser irradiation of 780 nm and different power levels (41.5 and 26.4 W/cm2). The ablation of local tumor cells irradiated by the laser was observed, and the greater the laser irradiation power is, the more tumor cells died (Choi et al., 2011).

### Combination of PTT and Chemotherapy

Drug resistance and system toxicity are the major shortcomings of chemotherapy (Peer et al., 2007). However, irradiation of NIR light not only causes a local temperature increase but also promotes the release of drugs in the nanocomposite. Therefore, the photothermal property of GNRs also exhibits the effect of drug release control, which allows GNRs and chemotherapeutic drugs to be incorporated in the same system, where the dual effects of high temperature and chemical drugs can kill tumor cells and enhance the therapeutic effect. Light-triggered drug release avoids the contact of chemotherapeutic drugs with normal tissues, and its efficient release at the treatment site also reduces the dosage of drugs used, thus minimizing the occurrence of dose-dependent side effects (Li Y. et al., 2018). For example, Liao et al. constructed a system of co-carrying GNRs and DOX, which achieved GNRs-mediated photothermal conversion and DOX light-triggered drug release under laser irradiation. The results showed that the combination therapy was better than PTT or chemotherapy alone. It was found that the dose of DOX in the combined treatment group was reduced by 50%, so the occurrence of side effects was reduced (Liao et al., 2015). The controlled drug release based on GNRs is a promising field, and more and more research is devoted to developing novel drug release systems. In the following paragraphs, several kinds of controlled drug release systems for GNRs based on PTT combined with chemotherapy are introduced.

#### Drug Release With pH/NIR Dual Response

Generally speaking, chemotherapeutic drugs loaded on the GNRs surface by encapsulation or physical adsorption often lead to unsatisfactory release behavior, which in turn leads to uncertainty in cancer treatment (Chen et al., 2014). Nanocomposites relying on endocytosis to enter cells are frequently trapped in lysosomal vesicles, resulting in the insufficient or slow intracellular release of drugs, as well as hindering its antitumor effect, especially in multidrug-resistant cells. It is easier to pump out little drugs with a more active efflux pump. Therefore, pH-sensitive nanocomposites can respond to pH gradients in lysosomes, thus promoting lysosomal escape to rapid intracellular release of drugs (Li W.-Q. et al., 2018). The following are the preparation process of some pH/NIR dual-response drug release systems.

Hou et al. synthesized a novel pH-sensitive targeted polysaccharide-GNRs conjugate carrying DOX through acidic unstable bonds. The pH decrease accelerated DOX release, confirming that the acid-induced hydrazine bond breakage promoted DOX release. It can prevent drug leakage while maintaining good stability under normal physiological conditions but trigger drug release rapidly in lysosomes and effectively reduce the side effects of leaky drugs, thus improving the therapeutic effect (Hou et al., 2019b). However, this novel nanomaterial can only load a relatively low dose of DOX and needs further improvement.

As a pH-sensitive switch, pH-responsive polyhistidine (PHIS), due to its unique properties and good biocompatibility, can be introduced into nanocomposites (Lee et al., 2003). Jiang et al. combined PHIS with mesoporous silica (mSiO_2_)-wrapped GNRs as known as GNRs/mSiO_2_. Because mSiO_2_ provides a specific area on the surface sufficient for drug loading, GNRs/mSiO_2_ have been developed previously for the combination of chemotherapy and PTT (Guo et al., 2020; Guo et al., 2021; Li et al., 2021). The acid response release characteristics of this material can be attributed to the protonation of PHIS under acidic conditions. The molecular motion is improved by NIR light absorption and light-to-heat energy conversion. Meanwhile, the weak bond between DOX and the hydroxyl groups of silica induces the rapid release of more DOX molecules. It is proven that the pH/NIR dually triggered drug release system of nanocomposites promotes intracellular drug release (Jiang et al., 2020).

Similarly, Xu et al. developed a pH/NIR dual-inspired drug release nanoplatform based on HA-modified GNRs. The nanoparticles have good stability and drug delivery behavior triggered by the pH and NIR, attributed to the heating of GNRs surrounding fluids caused by irradiation. The heating effect loosens the structure of the layer formed by HA and reduces the viscosity of the local solution of HA. Simultaneously, the heating effect promotes drug diffusion. In other words, the release of DOX can be adjusted by NIR irradiation and pH (Xu et al., 2017). Jin et al. synthesized GNRs/hydrogel core/shell nanospheres, and the release of 5-fluorouracil (5-FU) from nanospheres is significantly increased in mild acidic media. Moreover, the exposure to NIR light (808 nm) triggers a greater amount of 5-FU release (Jin et al., 2015).

#### Drug Release With Redox/pH/NIR Triple Response

S-S bonds in many nanomedicine delivery systems are often used in redox reactions, some of which can be used in combination with PTT, indicating that redox reaction release combined with hyperthermia is an effective method to inhibit tumors. For example, disulfide bonds are stable in circulation under physiological conditions. However, it can be cleaved quickly within cancer cells due to highly reductive environments (Zhang et al., 2019). The TME manifests the characteristics of low pH and high GSH level compared to normal tissue (Gao et al., 2014). To take advantage of this property, Hou et al. fabricated a GNRs-based complex containing hydrazine and disulfide bonds, which exhibits a pH/redox reaction dual-response drug release behavior (Hou et al., 2019a).

Similarly, some scholars combined GNRs with disulfiram (DSF), which exhibits GSH-, acid-, and laser-responsive release properties. DSFs have good stability with Au nanorods to avoid the premature release of drugs before reaching the tumor. This response release property makes it possible to selectively release drugs under internal and external stimuli, which provides a prospect for more efficient drug delivery (Xu et al., 2020).

A multifunctional nanosheet based on manganese dioxide (MnO_2_) and GNRs has been prepared in previous studies, in which MnO_2_ nanosheets are responsive to the slightly acidic environment and GSH-based reduction reaction, resulting in MnO_2_ degradation to manganese ions, leading to drug release (Gao et al., 2014). Wang et al. prepared PLGA nanoparticles loaded with GNRs and DTX (PLGA/AuNR/DTX) and then coated the ultra-thin nanofilm with MnO_2_ on the surface of it, constructing a new drug delivery system. Radiofrequency heating and MnO_2_ degradation can significantly promote controlled drug release in tumor regions (Wang et al., 2017).

All in all, combined with the unique response of GNRs to NIR, recent strategies depend on the breaking of bonds or denaturation of groups caused by acid, GSH, or other stimuli. More and more research has been devoted to developing special controlled drug release systems.

### Combination of PTT and Photodynamic Therapy (PDT)

PDT relies on reactive oxygen species (ROS) generated by photosensitizers under excitation light to kill tumor cells. Both PTT and PDT rely on external laser irradiation to activate the photosensitizer, which achieves good temporal and spatial control. Chuang et al. loaded the PTT agent GNRs and PDT agent Chlorine6 (Ce6) together in adipose-derived stem cells and gave irradiation at 808 and 660 nm to stimulate the conversion of heat energy and the formation of ROS, respectively. Compared to the control group, there was a higher tumor cell mortality rate (Chuang et al., 2020). Tham et al. used zinc phthalocyanine to produce singlet oxygen, and GNRs was used as a photothermal conversion agent. The high temperature can promote blood flow and attract more oxygen for ROS generation, thus promoting the oxidation of tumor cells, which indicates that the two types of therapy can behave synergistically. (Tham et al., 2016).

### Combination of PTT and Other Therapies

**FIGURE 7:**
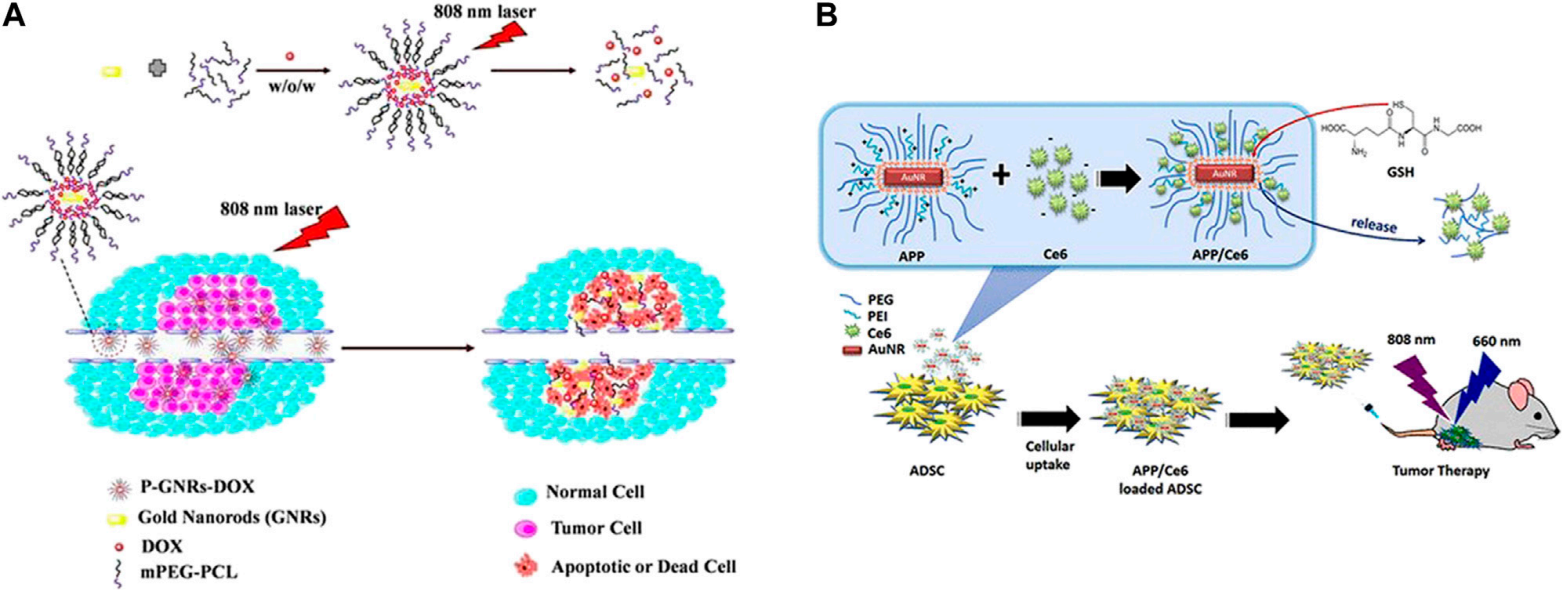
Combination of photothermal therapy and other therapies. **(A)** The combination of photothermal and chemotherapy using GNRs and doxorubicin co-loaded polymersomes (P-GNRs-DOX) irradiated by 808 nm laser. (Combined Cancer Photothermal-Chemotherapy Based on Doxorubicin/Gold Nanorod-Loaded Polymersomes, http://creativecommons.org/licenses/by-nc-nd/3.0/). **(B)** The combination of photothermal and photodynamic therapy using AuNR-PEG-PEI (APP)/chlorin e6 (Ce6)-loaded adipose-derived stem cell (ADSC) system irradiated by 808 nm light for PDT and 660 nm light for PTT. (Chuang et al., 2020).

In addition to chemotherapy and PDT (Figure 7; Chuang et al., 2020; Liao et al., 2015), other treatment methods, including RT and immunotherapy, can also be combined with PTT. Zhang et al. combined PTT and immunotherapy. The team constructed a GMPF-indoleamine 2,3-dioxygenase (IDO) small interfering RNA (siIDO) composite system, in which GNRs is used as a PTT agent, siIDO is used to induce antitumor immunity, and FA is used as a targeting medium. IDO is an immunosuppressive factor that can weaken the body’s antitumor immunity. This system uses siIDO to silence gene expression to achieve the effect of immunotherapy (Zhang et al., 2020). Although the specificity of PTT is greatly improved, damage to surrounding tissues can be inevitable and trigger certain inflammatory reactions. Therefore, Dong et al. combined PTT with anti-inflammatory therapy, in which they enwrapped aspirin and GNRs together, and showed a better effect (Dong et al., 2018).

## Discussion and Perspectives

To meet the need for cancer therapy, GNRs should possess less toxicity, higher yield, good monodispersity, and specific sizes, shapes, and ARs to achieve ideal optical properties, which puts forward more requirements in the production process. The common synthesis methods and surface functionalization of GNRs for PTT have been reviewed. Up to the present, the seed-mediated growth method has become the most widely used method for the synthesis of GNRs due to its advantages of long-run development and ease of AR control. Many researchers improved the characters of the final products by adjusting the parameters during the synthetic process. For instance, repeated cleaning and changing a less toxic surfactant such as C12EDMAB (Allen et al., 2017) could effectively reduce the cytotoxicity. The ARs, sizes, shapes, monodispersity, and yield of GNRs vary when the synthetic environment is changed, including temperature, pH, time, and concentration.

However, there are still scientific research debates on the best match for shape and size. For instance, gold nanoparticles with rod-like structures have the SPR effect, thus manifesting a better photothermal conversion effect. With increasing AR, the maximum absorption peak of GNRs also shows a redshift. The size of GNRs is a matter of great concern. It has been reviewed that as the size of GNRs decreases, the heat generation efficiency, blood retention, and intratumoral penetration could be improved. However, smaller GNRs also exhibited greater toxicity due to their high surface area compared to the mass and higher probability to interact with normal tissues. Therefore, smaller GNRs, while showing better therapeutic effects, also increase the potential for toxic side effects (Ali et al., 2019), which is a significant clinic concern. Although the seedless synthesis method did not initially attract much attention, some researchers used the method to acquire products that are difficult to obtain by traditional seed-mediated growth methods, such as small-sized GNRs. However, a more advanced method is needed to synthesize ultrasmall GNRs, as traditional technology tends to increase the concentration of seeds added in the growth solution, which sacrifices both yield and monodispersity (Chang and Murphy 2018; Cheng et al., 2019; Mbalaha et al., 2019).

Prolonging blood circulation time, increasing active or passive targeting in the tumor region, enhancing cell uptake, reducing systemic toxicity, and enhancing the photothermal effect or tumor-killing efficiency combined with other effects in the tumor region are the key points to improve the tumor-killing effect of GNRs, and these links are inseparable and interactive. Using stealth coating or other surface functionalization, reducing the size of material can reduce the interaction between the material and the blood system or other tissue systems and prolong the blood circulation time of GNRs material. Many studies have taken the two aspects into account: prolonged blood circulation and increased endocytosis. However, the PTT of GNRs is a coherent process. Thus, further exploration is needed in terms of specific mechanisms. Another debate is that coating polymers cannot exhibit all the important properties needed, and most of them possess only one or two of the required properties. Some of the properties needed are contradictory. Therefore, we need to balance these factors to achieve the optimal tumor-killing effect.

Due to various parameters, such as the cell type (Zhu et al., 2014; Mahmoud N. N. et al., 2019; Hashemi et al., 2019), medium component, incubation time, and dosage affecting cellular uptake, contradictory results occurred when studying the influence of surface chemical factors on cell uptake. In addition, the cellular uptake mechanism of GNRs and the interaction between nanorods and the biosphere are still poorly understood. Meanwhile, most studies on nanomaterials are conducted *in vitro*, unlike the real situation *in vivo* (Dobrovolskaia and McNeil 2007). Moreover, GNRs with a high cellular uptake rate usually have a better therapeutic effect but may lead to cytotoxicity sometimes. As cell uptake is mainly influenced by surface coatings, it is necessary to further compare and explore the uptake process of tumor cells with different ligands to prepare optimized GNRs for PTT.

PTT still requires more experimental research, and the long-term cytotoxicity of nanomaterials must be understood before it is fully applied in the clinic. Meanwhile, the mechanism of PTT-mediated cell death is complex. Besides cell injury caused by high temperature, other factors that may induce further effects on cell growth remain unknown, which is worthy of further exploration. It is believed that, through this review, more research will solve the above problems and lay a solid road for the clinical application of GNRs.

## Glossary

5-FU: 5-Fluorouracil
AA: ascorbic acid
AR: aspect ratio
BSA: bull serum albumin
CAIX: carbonic anhydrase IX
Ce6: Chlorine6
CIK cells: cytokine-induced killer cells
CPT: camptothecin
CS: chitosan
CXCR4: chemokine C-X-C-Motif receptor 4.
DOX: Doxorubicin
DSF: disulfiram
dSiO2: dense silica
ECM: extracellular matrix
EPPT: Glu-Pro-Pro-Thr
EPR effects: enhanced permeability and retention effect
FA: folic acid
GMPF: GNR-MUA-PEI-FA
GNRs: gold nanorods
GNRs: gold nanorods
GSSG: oxidized glutathione
HA: hyaluronic acid
HAS: human serum albumin
IDO: Indoleamine 2,3-dioxygenase-1
IFP: interstitial fluid pressure
iPS cells: induced pluripotent stem cells
KMnO4: potassium permanganate
LSPR: longitudinal surface plasmon resonance
MDR: multiple resistant bacteria
MMP-9: matrix metalloproteinase-9; TPP
MnO2: manganese dioxide
MPAP: myristoylated polyarginine peptide
mSiO2: mesoporous silica
MUA: Mercaptoun-decanoic acid
NIR: near infrared ray
PC: protein corona; PEG
PCL: poly (ε-caprolactone)
PDT: photodynamic therapy
PEI: polyethyleneimine
PHIS: PH responsive polyhistidine
PLGA: poly (lactic-co-glycolic acid)
PEG: poly (ethylene glycol)
PSS: poly (sodium 4-styrenesulfonate)
PTT: photothermal therapy
RGD: arginylglycylaspartic acid
RME: receptor-mediated endocytosis
ROS: reactive oxygen species
RT: radiation therapy
SA: sialic acid; HCl, hydrochloric acid
SDT: sonodynamic therapy
TiO2: titanium dioxide
TME: tumor microenvironment
TPP: triphenylphosphonium bromide

## References

[B1] Abdelrasoul G. N. Magrassi R. Dante S. d’Amora M. d’Abbusco M. S. Pellegrino T. (2016). PEGylated gold nanorods as optical trackers for biomedical applications: anin vivoandin vitrocomparative study. Nanotechnology 27, 255101. 10.1088/0957-4484/27/25/255101 27176116

[B2] Adamiak L. Touve M. A. LeGuyader C. L. M. Gianneschi N. C. (2017). Peptide brush polymers and nanoparticles with enzyme-regulated structure and charge for inducing or evading macrophage cell uptake. ACS Nano. 11, 9877–9888. 10.1021/acsnano.7b03686 28972735

[B3] Akhter M. H. Rizwanullah M. Ahmad J. Ahsan M. J. Mujtaba M. A. Amin S. (2018). Nanocarriers in advanced drug targeting: setting novel paradigm in cancer therapeutics. Artif. Cell Nanomedicine, Biotechnol. 46, 873–884. 10.1080/21691401.2017.1366333 28830262

[B4] Ali M. R. K. Rahman M. A. Wu Y. Han T. Peng X. Mackey M. A. (2017a). Efficacy, long-term toxicity, and mechanistic studies of gold nanorods photothermal therapy of cancer in xenograft mice. Proc. Natl. Acad. Sci. USA. 114, E3110–E3118. 10.1073/pnas.1619302114 28356516 PMC5393247

[B5] Ali M. R. K. Rahman M. A. Wu Y. Han T. Peng X. Mackey M. A. (2017b). Efficacy, long-term toxicity, and mechanistic studies of gold nanorods photothermal therapy of cancer in xenograft mice. Proc. Natl. Acad. Sci. USA. 114, E3110–e3118. 10.1073/pnas.1619302114 28356516 PMC5393247

[B6] Ali M. R. K. Snyder B. El-Sayed M. A. (2012). Synthesis and optical properties of small Au nanorods using a seedless growth technique. Langmuir. 28, 9807–9815. 10.1021/la301387p 22620850

[B7] Ali M. R. K. Wu Y. El-Sayed M. A. (2019). Gold-nanoparticle-assisted plasmonic photothermal therapy advances toward clinical application. J. Phys. Chem. C 123, 15375–15393. 10.1021/acs.jpcc.9b01961

[B8] Ali M. R. K. Wu Y. Tang Y. Xiao H. Chen K. Han T. (2017c). Targeting cancer cell integrins using gold nanorods in photothermal therapy inhibits migration through affecting cytoskeletal proteins. Proc. Natl. Acad. Sci. USA. 114, E5655–e5663. 10.1073/pnas.1703151114 28652358 PMC5514737

[B9] Alkilany A. M. Murphy C. J. (2010). Toxicity and cellular uptake of gold nanoparticles: what we have learned so far? J. Nanopart Res. 12, 2313–2333. 10.1007/s11051-010-9911-8 21170131 PMC2988217

[B10] Alkilany A. M. Nagaria P. K. Hexel C. R. Shaw T. J. Murphy C. J. Wyatt M. D. (2009). Cellular uptake and cytotoxicity of gold nanorods: molecular origin of cytotoxicity and surface effects. Small. 5, 701–708. 10.1002/smll.200801546 19226599

[B11] Alkilany A. M. Shatanawi A. Kurtz T. Caldwell R. B. Caldwell R. W. (2012). Toxicity and cellular uptake of gold nanorods in vascular endothelium and smooth muscles of isolated rat blood vessel: importance of surface modification. Small 8, 1270–1278. 10.1002/smll.201101948 22334586 PMC3798057

[B12] Allen J. M. Xu J. Blahove M. Canonico-May S. A. Santaloci T. J. Braselton M. E. (2017). Synthesis of less toxic gold nanorods by using dodecylethyldimethylammonium bromide as an alternative growth-directing surfactant. J. Colloid Interf. Sci. 505, 1172–1176. 10.1016/j.jcis.2017.06.101 28715861

[B13] An L. Wang Y. Tian Q. Yang S. (2017). Small gold nanorods: recent advances in synthesis, biological imaging, and cancer therapy. Materials (Basel, Switzerland). 10, 1372. 10.3390/ma10121372 PMC5744307 29189739

[B14] An L. Wang Y. Lin J. Tian Q. Xie Y. Hu J. (2019). Macrophages-mediated delivery of small gold nanorods for tumor hypoxia photoacoustic imaging and enhanced photothermal therapy. ACS Appl. Mater. Inter. 11, 15251–15261. 10.1021/acsami.9b00495 30964253

[B15] Bagley A. F. Hill S. Rogers G. S. Bhatia S. N. (2013). Plasmonic photothermal heating of intraperitoneal tumors through the use of an implanted near-infrared source. ACS nano. 7, 8089–8097. 10.1021/nn4033757 23961973 PMC3788585

[B16] Bandyopadhyay S. McDonagh B. H. Singh G. Raghunathan K. Sandvig A. Sandvig I. (2018). Growing gold nanostructures for shape-selective cellular uptake. Nanoscale Res. Lett. 13, 254. 10.1186/s11671-018-2662-7 30155798 PMC6113194

[B17] Beere H. M. (2004). `The stress of dying': the role of heat shock proteins in the regulation of apoptosis. J. Cel. Sci. 117, 2641–2651. 10.1242/jcs.01284 15169835

[B18] Bertrand N. Wu J. Xu X. Kamaly N. Farokhzad O. C. (2014). *Cancer* nanotechnology: the impact of passive and active targeting in the era of modern cancer biology. Adv. Drug Deliv. Rev. 66, 2–25. 10.1016/j.addr.2013.11.009 24270007 PMC4219254

[B19] Bhana S. O’Connor R. Johnson J. Ziebarth J. D. Henderson L. Huang X. (2016). Photosensitizer-loaded gold nanorods for near infrared photodynamic and photothermal cancer therapy. J. Colloid Interf. Sci. 469, 8–16. 10.1016/j.jcis.2016.02.012 26866884

[B20] Black K. C. L. Wang Y. Luehmann H. P. Cai X. Xing W. Pang B. (2014). Radioactive 198Au-doped nanostructures with different shapes for *in vivo* analyses of their biodistribution, tumor uptake, and intratumoral distribution. ACS Nano. 8, 4385–4394. 10.1021/nn406258m 24766522 PMC4358630

[B21] Boca S. C. Potara M. Gabudean A.-M. Juhem A. Baldeck P. L. Astilean S. (2011). Chitosan-coated triangular silver nanoparticles as a novel class of biocompatible, highly effective photothermal transducers for *in vitro* cancer cell therapy. Cancer Lett. 311, 131–140. 10.1016/j.canlet.2011.06.022 21840122

[B22] Borri C. Centi S. Ratto F. Pini R. (2018). Polylysine as a functional biopolymer to couple gold nanorods to tumor-tropic cells. J. Nanobiotechnology 16, 50. 10.1186/s12951-018-0377-7 29855304 PMC5984317

[B23] Bose R. J. Paulmurugan R. Moon J. Lee S.-H. Park H. (2018). Cell membrane-coated nanocarriers: the emerging targeted delivery system for cancer theranostics. Drug Discov. Today 23, 891–899. 10.1016/j.drudis.2018.02.001 29426004

[B24] Bray F. Ferlay J. Soerjomataram I. Siegel R. L. Torre L. A. Jemal A. (2018). Global cancer statistics 2018: GLOBOCAN estimates of incidence and mortality worldwide for 36 cancers in 185 countries. CA: A Cancer J. Clinicians. 68, 394–424. 10.3322/caac.21492 30207593

[B25] Burrows N. D. Harvey S. Idesis F. A. Murphy C. J. (2017). Understanding the seed-mediated growth of gold nanorods through a fractional factorial design of experiments. Langmuir. 33, 1891–1907. 10.1021/acs.langmuir.6b03606 27983861

[B26] Cai K. Zhang W. Zhang J. Li H. Han H. Zhai T. (2018). Design of gold hollow nanorods with controllable aspect ratio for multimodal imaging and combined chemo-photothermal therapy in the second near-infrared window. ACS Appl. Mater. Inter. 10, 36703–36710. 10.1021/acsami.8b12758 30284807

[B27] Chang H.-H. Murphy C. J. (2018). Mini gold nanorods with tunable plasmonic peaks beyond 1000 nm. Chem. Mater. 30, 1427–1435. 10.1021/acs.chemmater.7b05310 31404258 PMC6688645

[B28] Chen H. Xiao L. Anraku Y. Mi P. Liu X. Cabral H. (2014). Polyion complex vesicles for photoinduced intracellular delivery of amphiphilic photosensitizer. J. Am. Chem. Soc. 136, 157–163. 10.1021/ja406992w 24283288

[B29] Chen R. Wang X. Yao X. Zheng X. Wang J. Jiang X. (2013). Near-IR-triggered photothermal/photodynamic dual-modality therapy system via chitosan hybrid nanospheres. Biomaterials 34, 8314–8322. 10.1016/j.biomaterials.2013.07.034 23896004

[B30] Chen Y. Bian X. Aliru M. Deorukhkar A. A. Ekpenyong O. Liang S. (2018a). Hypoxia-targeted gold nanorods for cancer photothermal therapy. Oncotarget. 9, 26556–26571. 10.18632/oncotarget.25492 29899876 PMC5995181

[B31] Chen Y. Bian X. Aliru M. Deorukhkar A. A. Ekpenyong O. Liang S. (2018b). Hypoxia-targeted gold nanorods for cancer photothermal therapy. Oncotarget. 9, 26556–26571. 10.18632/oncotarget.25492 29899876 PMC5995181

[B32] Cheng M. J. Bal N. N. Prabakaran P. Kumar R. Webster T. J. Sridhar S. (2019). Ultrasmall gold nanorods: synthesis and glycocalyx-related permeability in human endothelial cells. Int J Nanomedicine Vol. 14, 319–333. 10.2147/IJN.S184455 PMC6340363 30697044

[B33] Chithrani B. D. Ghazani A. A. Chan W. C. W. (2006). Determining the size and shape dependence of gold nanoparticle uptake into mammalian cells. Nano Lett. 6, 662–668. 10.1021/nl052396o 16608261

[B34] Chithrani D. B. (2010). Intracellular uptake, transport, and processing of gold nanostructures. Mol. Membr. Biol. 27, 299–311. 10.3109/09687688.2010.507787 20929337

[B35] Choi J. Lee S.-E. Park J.-S. Kim S. Y. (2018). Gold nanorod-photosensitizer conjugates with glutathione-sensitive linkages for synergistic cancer photodynamic/photothermal therapy. Biotechnol. Bioeng. 115, 1340–1354. 10.1002/bit.26536 29288576

[B36] Choi W. I. Kim J.-Y. Kang C. Byeon C. C. Kim Y. H. Tae G. (2011). Tumor regression *in vivo* by photothermal therapy based on gold-nanorod-loaded, functional nanocarriers. ACS nano. 5, 1995–2003. 10.1021/nn103047r 21344891

[B37] Chu D. Dong X. Zhao Q. Gu J. Wang Z. (2017). Photosensitization priming of tumor microenvironments improves delivery of nanotherapeutics via neutrophil infiltration. Adv. Mater. Weinheim 29. 10.1002/adma.201701021 PMC5510494 28504320

[B38] Chuang C.-C. Chen Y.-N. Wang Y.-Y. Huang Y.-C. Lin S.-Y. Huang R.-Y. (2020). Stem Cell-Based Delivery of Gold/Chlorin e6 Nanocomplexes for Combined Photothermal and Photodynamic Therapy. ACS Appl. Mater. Inter. 12, 30021–30030. 10.1021/acsami.0c03446 32594734

[B39] Chuang C. C. Cheng C. C. Chen P. Y. Lo C. Chen Y. N. Shih M. H. (2019). Gold nanorod-encapsulated biodegradable polymeric matrix for combined photothermal and chemo-cancer therapy. Int. J. Nanomedicine 14, 181–193. 10.2147/IJN.S177851 30613145 PMC6306055

[B40] Davis M. E. Chen Z. Shin D. M. (2008). Nanoparticle therapeutics: an emerging treatment modality for cancer. Nat. Rev. Drug Discov. 7, 771–782. 10.1038/nrd2614 18758474

[B41] Ding L. Yao C. Yin X. Li C. Huang Y. Wu M. (2018). Size, shape, and protein corona determine cellular uptake and removal mechanisms of gold nanoparticles. Small 14, e1801451. 10.1002/smll.201801451 30239120

[B42] Dobrovolskaia M. A. McNeil S. E. (2007). Immunological properties of engineered nanomaterials. Nat. Nanotech. 2, 469–478. 10.1038/nnano.2007.223 18654343

[B43] Dong Q. Wang X. Hu X. Xiao L. Zhang L. Song L. (2018). Simultaneous application of photothermal therapy and an anti-inflammatory prodrug using pyrene-aspirin-loaded gold nanorod graphitic nanocapsules. Angew. Chem. 130, 183–187. 10.1002/ange.201709648 29125675

[B44] Du Y. Jiang Q. Beziere N. Song L. Zhang Q. Peng D. (2016a). DNA-Nanostructure-Gold-Nanorod hybrids for enhanced in vivo optoacoustic imaging and photothermal therapy. Adv. Mater. 28, 10000–10007. 10.1002/adma.201601710 27679425

[B45] Du Y. Jiang Q. Beziere N. Song L. Zhang Q. Peng D. (2016b). DNA-Nanostructure-Gold-Nanorod hybrids for enhanced in vivo optoacoustic imaging and photothermal therapy. Adv. Mater. 28, 10000–10007. 10.1002/adma.201601710 27679425

[B46] Encinas-Basurto D. Ibarra J. Juarez J. Pardo A. Barbosa S. Taboada P. (2018). Hybrid folic acid-conjugated gold nanorods-loaded human serum albumin nanoparticles for simultaneous photothermal and chemotherapeutic therapy. Mater. Sci. Eng. C. 91, 669–678. 10.1016/j.msec.2018.06.002 30033301

[B47] Favi P. M. Valencia M. M. Elliott P. R. Restrepo A. Gao M. Huang H. (2015). Shape and surface chemistry effects on the cytotoxicity and cellular uptake of metallic nanorods and nanospheres. J. Biomed. Mater. Res. 103, 3940–3955. 10.1002/jbm.a.35518 26053238

[B48] Fu G. Liu W. Feng S. Yue X. (2012). Prussian blue nanoparticles operate as a new generation of photothermal ablation agents for cancer therapy. Chem. Commun. 48, 11567–11569. 10.1039/c2cc36456e 23090583

[B49] Gallina M. E. Zhou Y. Johnson C. J. Harris-Birtill D. Singh M. Zhao H. (2016). Aptamer-conjugated, fluorescent gold nanorods as potential cancer theradiagnostic agents. Mater. Sci. Eng. C. 59, 324–332. 10.1016/j.msec.2015.09.101 26652380

[B50] Gao B. Xu J. He k.-W. Shen L. Chen H. Yang H.-J. (2016). Cellular uptake and intra-organ biodistribution of functionalized silica-coated gold nanorods. Mol. Imaging Biol. 18, 667–676. 10.1007/s11307-016-0938-9 26884056

[B51] Gao C. Liu T. Dang Y. Yu Z. Wang W. Guo J. (2014). pH/redox responsive core cross-linked nanoparticles from thiolated carboxymethyl chitosan for *in vitro* release study of methotrexate. Carbohydr. Polym. 111, 964–970. 10.1016/j.carbpol.2014.05.012 25037437

[B52] García I. Henriksen-Lacey M. Sánchez-Iglesias A. Grzelczak M. Penadés S. Liz-Marzán L. M. (2015). Residual CTAB ligands as mass Spectrometry labels to monitor cellular uptake of Au nanorods. J. Phys. Chem. Lett. 6, 2003–2008. 10.1021/acs.jpclett.5b00816 26266492

[B53] Ghosh P. Thambi V. Khatua S. Chakraborty A. L. (2017). Synthesis of gold nanorods with tunable surface plasmon resonance for near-infrared biosensing applications. 2017 IEEE Workshop on Recent Advances in Photonics (WRAP), Hyderabad, India, September 20, 2018 (New York, NY: IEEE), 1–3. 10.1109/WRAP.2017.8468598

[B54] Golubev A. A. Prilepskii A. Y. Dykman L. A. Khlebtsov N. G. Bogatyrev V. A. (2016). Colorimetric evaluation of the viability of the MicroalgaDunaliella salinaas a test tool for nanomaterial toxicity. Toxicol. Sci. 151, 115–125. 10.1093/toxsci/kfw023 26865664 PMC4914795

[B55] González-Rubio G. Kumar V. Llombart P. Díaz-Núñez P. Bladt E. Altantzis T. (2019). Disconnecting symmetry breaking from seeded growth for the reproducible synthesis of high quality gold nanorods. ACS Nano. 13, 4424–4435. 10.1021/acsnano.8b09658 30939242

[B56] Guo D. Huang Y. Jin X. Zhang C. Zhu X. (2020). A redox-responsive, in-situ polymerized polyplatinum(IV)-Coated gold nanorod as an amplifier of tumor accumulation for enhanced thermo-chemotherapy. Biomaterials 266, 120400. 10.1016/j.biomaterials.2020.120400 33022477

[B57] Guo H. Liu X. Gui R. Wang Z. (2015). Facile synthesis of gold nanorods/hydrogels core/shell nanospheres for pH and near-infrared-light induced release of 5-fluorouracil and chemo-photothermal therapy. Colloids Surf. B: Biointerfaces 128, 498–505. 10.1016/j.colsurfb.2015.02.049 25794443

[B58] Guo H. Yi S. Feng K. Xia Y. (2021). *In situ* formation of metal organic framework onto gold nanorods/mesoporous silica with functional integration for targeted theranostics. Chem. Eng. J. 403, 126432. 10.1016/j.cej.2020.126432

[B59] Habash R. W. Bansal R. Krewski D. Alhafid H. T. (2006). Thermal therapy, part 1: an introduction to thermal therapy. Crit. Reviews™ Biomed. Eng. 34, 459-89. 10.1615/critrevbiomedeng.v34.i6.20 17725479

[B60] Haine A. T. Niidome T. (2017). Gold nanorods as nanodevices for bioimaging, photothermal therapeutics, and drug delivery. Chem. Pharm. Bull. 65, 625–628. 10.1248/cpb.c17-00102 28674334

[B61] Han H. Valdepérez D. Jin Q. Yang B. Li Z. Wu Y. (2017). Dual enzymatic reaction-assisted gemcitabine delivery systems for programmed pancreatic cancer therapy. ACS Nano. 11, 1281–1291. 10.1021/acsnano.6b05541 28071891

[B62] Han S.-S. Li Z.-Y. Zhu J.-Y. Han K. Zeng Z.-Y. Hong W. (2015). Dual-pH sensitive charge-reversal polypeptide micelles for tumor-triggered targeting uptake and nuclear drug delivery. Small 11, 2543–2554. 10.1002/smll.201402865 25626995

[B63] Hanahan D. Weinberg R. A. (2011). Hallmarks of cancer: the next generation. Cell. 144, 646–674. 10.1016/j.cell.2011.02.013 21376230

[B64] Hashemi F. Hormozi-Nezhad M. R. Corbo C. Farvadi F. Shokrgozar M. A. Mehrjoo M. (2019). Laser irradiation affects the biological identity and cellular uptake of plasmonic nanoparticles. Nanoscale 11, 5974–5981. 10.1039/c8nr09622h 30892307

[B65] He J. Unser S. Bruzas I. Cary R. Shi Z. Mehra R. (2018). The facile removal of CTAB from the surface of gold nanorods. Colloids Surf. B: Biointerfaces 163, 140–145. 10.1016/j.colsurfb.2017.12.019 29291499

[B66] He J. Zheng W. Ligmajer F. Chan C.-F. Bao Z. Wong K.-L. (2017). Plasmonic enhancement and polarization dependence of nonlinear upconversion emissions from single gold nanorod@SiO(2)@CaF(2):Yb(3+),Er(3+) hybrid core-shell-satellite nanostructures. Light Sci. Appl. 6, e16217. 10.1038/lsa.2016.217 30167245 PMC6062198

[B67] Hormozi-Nezhad M. R. Robatjazi H. Jalali-Heravi M. (2013). Thorough tuning of the aspect ratio of gold nanorods using response surface methodology. Anal. Chim. Acta 779, 14–21. 10.1016/j.aca.2013.03.056 23663667

[B68] Hossen S. Hossain M. K. Basher M. K. Mia M. N. H. Rahman M. T. Uddin M. J. (2019). Smart nanocarrier-based drug delivery systems for cancer therapy and toxicity studies: a review. J. Adv. Res. 15, 1–18. 10.1016/j.jare.2018.06.005 30581608 PMC6300464

[B69] Hou G. Qian J. Xu W. Sun T. Wang J. Wang Y. (2019a). Multifunctional PEG-b-polypeptide-decorated gold nanorod for targeted combined chemo-photothermal therapy of breast cancer. Colloids Surf. B: Biointerfaces 181, 602–611. 10.1016/j.colsurfb.2019.05.025 31202131

[B70] Hou G. Qian J. Xu W. Sun T. Wang Y. Wang J. (2019b). A novel pH-sensitive targeting polysaccharide-gold nanorod conjugate for combined photothermal-chemotherapy of breast cancer. Carbohydr. Polym. 212, 334–344. 10.1016/j.carbpol.2019.02.045 30832865

[B71] Huang C.-J. Chiu P.-H. Wang Y.-H. Yang C.-F. (2006). Synthesis of the gold nanodumbbells by electrochemical method. J. Colloid Interf. Sci. 303, 430–436. 10.1016/j.jcis.2006.07.073 16930612

[B72] Huang H. Li H. Wang H. Li J. Li P. Chen Q. (2018). Morphological control of gold nanorods via thermally driven bi-surfactant growth and application for detection of heavy metal ions. Nanotechnology 29, 334001. 10.1088/1361-6528/aac6b2 29786615

[B73] Huang X. Peng X. Wang Y. Wang Y. Shin D. M. El-Sayed M. A. (2010). A reexamination of active and passive tumor targeting by using rod-shaped gold nanocrystals and covalently conjugated peptide ligands. ACS Nano. 4, 5887–5896. 10.1021/nn102055s 20863096 PMC2964428

[B74] Huang Y. Xia K. He N. Lu Z. Zhang L. Deng Y. (2015). Size-tunable synthesis of gold nanorods using pyrogallol as a reducing agent. Chemistry 58, 1759–1765. 10.1007/s11426-015-5437-3

[B75] Huff T. B. Hansen M. N. Zhao Y. Cheng J.-X. Wei A. (2007). Controlling the cellular uptake of gold nanorods. Langmuir. 23, 1596–1599. 10.1021/la062642r 17279633 PMC2818780

[B76] Hughes D. (2003). Exploiting genomics, genetics and chemistry to combat antibiotic resistance. Nat. Rev. Genet. 4, 432–441. 10.1038/nrg1084 12776213

[B77] Hussein E. Zagho M. Nasrallah G. Elzatahry A. (2018). Recent advances in functional nanostructures as cancer photothermal therapy. Int J Nanomedicine Vol. 13, 2897–2906. 10.2147/ijn.s161031 PMC5961635 29844672

[B78] Hwang S. Nam J. Jung S. Song J. Doh H. Kim S. (2014). Gold nanoparticle-mediated photothermal therapy: current status and future perspective. Nanomedicine 9, 2003–2022. 10.2217/nnm.14.147 25343350

[B79] Ishizawa T. Fukushima N. Shibahara J. Masuda K. Tamura S. Aoki T. (2009). Real-time identification of liver cancers by using indocyanine green fluorescent imaging. Cancer 115, 2491–2504. 10.1002/cncr.24291 19326450

[B80] Jana N. R. Gearheart L. Murphy C. J. (2001). Seed-mediated growth approach for shape-controlled synthesis of spheroidal and rod-like gold nanoparticles using a surfactant template. Adv. Mater. 13, 1389–1393. 10.1002/1521-4095(200109)13:18<1389::aid-adma1389>3.0.co;2-f-F

[B81] Jana N. R. (2005). Gram-scale synthesis of soluble, near-monodisperse gold nanorods and other anisotropic nanoparticles. Small 1, 875–882. 10.1002/smll.200500014 17193542

[B82] Jessl S. Tebbe M. Guerrini L. Fery A. Alvarez-Puebla R. A. Pazos-Perez N. (2018). Silver-assisted synthesis of gold nanorods: the relation between silver additive and iodide impurities. Small 14, e1703879. 10.1002/smll.201703879 29665260

[B83] Jiang A. Liu Y. Ma L. Mao F. Liu L. Zhai X. (2019). Biocompatible heat-shock protein inhibitor-delivered flowerlike short-wave infrared nanoprobe for mild temperature-driven highly efficient tumor ablation. ACS Appl. Mater. Inter. 11, 6820–6828. 10.1021/acsami.8b21483 30677285

[B84] Jiang Q. Shi Y. Zhang Q. Li N. Zhan P. Song L. (2015). A self-assembled DNA origami-gold nanorod complex for cancer theranostics. Small 11, 5134–5141. 10.1002/smll.201501266 26248642

[B85] Jiang W. Kim B. Y. S. Rutka J. T. Chan W. C. W. (2008). Nanoparticle-mediated cellular response is size-dependent. Nat. Nanotech. 3, 145–150. 10.1038/nnano.2008.30 18654486

[B86] Jiang Y. Guo Z. Fang J. Wang B. Lin Z. Chen Z.-S. (2020). A multi-functionalized nanocomposite constructed by gold nanorod core with triple-layer coating to combat multidrug resistant colorectal cancer. Mater. Sci. Eng. C. 107, 110224. 10.1016/j.msec.2019.110224 31761194

[B87] Jin H. Liu X. Gui R. Wang Z. (2015). Facile synthesis of gold nanorods/hydrogels core/shell nanospheres for pHand near-infrared-light induced release of 5-fluorouracil and chemo-photothermal therapy. ColloidsSurf B Biointerfaces 128, 498–505. 10.1016/j.colsurfb.2015.02.049 25794443

[B88] Kah J. C. Y. Grabinski C. Untener E. Garrett C. Chen J. Zhu D. (2014). Protein coronas on gold nanorods passivated with amphiphilic ligands affect cytotoxicity and cellular response to penicillin/streptomycin. ACS Nano. 8, 4608–4620. 10.1021/nn5002886 24758495

[B89] Kam N. W. O'Connell M. Wisdom J. A. Dai H. (2005). Carbon nanotubes as multifunctional biological transporters and near-infrared agents for selective cancer cell destruction. Proc. Natl. Acad. Sci. USA. 102, 11600–11605. 10.1073/pnas.0502680102 16087878 PMC1187972

[B90] Kennedy L. C. Bickford L. R. Lewinski N. A. Coughlin A. J. Hu Y. Day E. S. (2011). A new era for cancer treatment: gold-nanoparticle-mediated thermal therapies. Small. 7, 169–183. 10.1002/smll.201000134 21213377

[B91] Khanal B. P. Zubarev E. R. (2019). Gram-scale synthesis of isolated monodisperse gold nanorods. Chem. Eur. J. 25, 1595–1600. 10.1002/chem.201805571 30471145

[B92] Khlebtsov B. N. Khanadeev V. A. Khlebtsov N. G. (2014a). Extinction and extra-high depolarized light scattering spectra of gold nanorods with improved purity and dimension tunability: direct and inverse problems. Phys. Chem. Chem. Phys. 16, 5710–5722. 10.1039/c3cp55414g 24522336

[B93] Khlebtsov B. N. Khanadeev V. A. Ye J. Sukhorukov G. B. Khlebtsov N. G. (2014b). Overgrowth of gold nanorods by using a binary surfactant mixture. Langmuir. 30, 1696–1703. 10.1021/la404399n 24460392

[B94] Kim T. Y. Kim J.-H. Kim M. P. Yi G.-R. (2016). Anion-mediated end-shape control in seed-mediated growth of gold nanorods. J. Nanosci. Nanotechnol. 16, 6327–6331. 10.1166/jnn.2016.12144 27427712

[B95] Kola I. Landis J. (2004). Can the pharmaceutical industry reduce attrition rates?. Nat. Rev. Drug Discov. 3, 711–716. 10.1038/nrd1470 15286737

[B96] Kozek K. A. Kozek K. M. Wu W. C. Mishra S. R. Tracy J. B. (2013). Large-scale synthesis of gold nanorods through continuous secondary growth. Chem. Mater. 25. 10.1021/cm402277y PMC3883054 24415848

[B97] Kwizera E. A. O'Connor R. Vinduska V. Williams M. Butch E. R. Snyder S. E. (2018). Molecular detection and analysis of exosomes using surface-enhanced Raman scattering gold nanorods and a miniaturized device. Theranostics 8, 2722–2738. 10.7150/thno.21358 29774071 PMC5957005

[B98] Lai J. Zhang L. Niu W. Qi W. Zhao J. Liu Z. (2014). One-pot synthesis of gold nanorods using binary surfactant systems with improved monodispersity, dimensional tunability and plasmon resonance scattering properties. Nanotechnology 25, 125601. 10.1088/0957-4484/25/12/125601 24571958

[B99] Lal S. Clare S. E. Halas N. J. (2008). Nanoshell-enabled photothermal cancer therapy: impending clinical impact. Acc. Chem. Res. 41, 1842–1851. 10.1021/ar800150g 19053240

[B100] Lanneau D. Brunet M. Frisan E. Solary E. Fontenay M. Garrido C. (2008). Heat shock proteins: essential proteins for apoptosis regulation. J. Cell. Mol. Med. 12, 743–761. 10.1111/j.1582-4934.2008.00273.x 18266962 PMC4401125

[B101] Lee D.-E. Koo H. Sun I.-C. Ryu J. H. Kim K. Kwon I. C. (2012). Multifunctional nanoparticles for multimodal imaging and theragnosis. Chem. Soc. Rev. 41, 2656–2672. 10.1039/c2cs15261d 22189429

[B102] Lee E. S. Shin H. J. Na K. Bae Y. H. (2003). Poly(L-histidine)-PEG block copolymer micelles and pH-induced destabilization. J. Controlled Release. 90, 363–374. 10.1016/s0168-3659(03)00205-0 12880703

[B103] Lee Y. J. Ahn E. Y. Park Y. (2019). Shape-dependent cytotoxicity and cellular uptake of gold nanoparticles synthesized using green tea extract. Nanoscale Res. Lett. 14, 129. 10.1186/s11671-019-2967-1 30976946 PMC6459462

[B104] Li P. Wu Y. Li D. Su X. Luo C. Wang Y. (2018a). Seed-mediated synthesis of tunable-aspect-ratio gold nanorods for near-infrared photoacoustic imaging. Nanoscale Res. Lett. 13, 313. 10.1186/s11671-018-2734-8 30288620 PMC6172158

[B105] Li W.-Q. Sun L.-P. Xia Y. Hao S. Cheng G. Wang Z. (2018b). Preoccupation of empty carriers decreases endo-/lysosome escape and reduces the protein delivery efficiency of mesoporous silica nanoparticles. ACS Appl. Mater. Inter. 10, 5340–5347. 10.1021/acsami.7b18577 29345456

[B106] Li X. Hou Y. Meng X. Li G. Xu F. Teng L. (2021). Folate receptor-targeting mesoporous silica-coated gold nanorod nanoparticles for the synergistic photothermal therapy and chemotherapy of rheumatoid arthritis. RSC Adv. 11, 3567–3574. 10.1039/d0ra08689d PMC8694156 35424296

[B107] Li Y. He D. Tu J. Wang R. Zu C. Chen Y. (2018c). The comparative effect of wrapping solid gold nanoparticles and hollow gold nanoparticles with doxorubicin-loaded thermosensitive liposomes for cancer thermo-chemotherapy. Nanoscale. 10, 8628–8641. 10.1039/c7nr09083h 29697100

[B108] Li Y. Lu W. Huang Q. Li C. Chen W. (2010). Copper sulfide nanoparticles for photothermal ablation of tumor cells. Nanomedicine 5, 1161–1171. 10.2217/nnm.10.85 21039194

[B109] Liao J. Li W. Peng J. Yang Q. Li H. Wei Y. (2015). Combined cancer photothermal-chemotherapy based on doxorubicin/gold nanorod-loaded polymersomes. Theranostics 5, 345–356. 10.7150/thno.10731 25699095 PMC4329499

[B110] Liu K. Bu Y. Zheng Y. Jiang X. Yu A. Wang H. (2017a). Seedless synthesis of monodispersed gold nanorods with remarkably high yield: synergistic effect of template modification and growth kinetics regulation. Chem. Eur. J. 23, 3291–3299. 10.1002/chem.201605617 28074502

[B111] Liu P. Wang Y. Liu Y. Tan F. Li J. Li N. (2020). S-nitrosothiols loaded mini-sized Au@silica nanorod elicits collagen depletion and mitochondrial damage in solid tumor treatment. Theranostics 10, 6774–6789. 10.7150/thno.42661 32550903 PMC7295055

[B112] Liu X. Yao J. Luo J. Duan X. Yao Y. Liu T. (2017b). Effect of growth temperature on tailoring the size and aspect ratio of gold nanorods. Langmuir 33, 7479–7485. 10.1021/acs.langmuir.7b01635 28696699

[B113] Liu Y. Crawford B. M. Vo-Dinh T. (2018). Gold nanoparticles-mediated photothermal therapy and immunotherapy. Immunotherapy 10, 1175–1188. 10.2217/imt-2018-0029 30236026

[B114] Liu Y. Yang M. Zhang J. Zhi X. Li C. Zhang C. (2016). Human induced pluripotent stem cells for tumor targeted delivery of gold nanorods and enhanced photothermal therapy. ACS Nano. 10, 2375–2385. 10.1021/acsnano.5b07172 26761620

[B115] Liu Y. Zhao Y. Wang Y. Li C. M. (2015). Polyamine-capped gold nanorod as a localized surface Plasmon resonance probe for rapid and sensitive copper(II) ion detection. J. Colloid Interf. Sci. 439, 7–11. 10.1016/j.jcis.2014.10.023 25463169

[B116] Lohse S. E. Murphy C. J. (2013). The quest for shape control: a history of gold nanorod synthesis. Chem. Mater. 25, 1250–1261. 10.1021/cm303708p

[B117] Mahmoud N. N. Al-Kharabsheh L. M. Khalil E. A. Abu-Dahab R. (2019b). Interaction of gold nanorods with human dermal fibroblasts: cytotoxicity, cellular uptake, and wound healing. Nanomaterials (Basel). 9, 1131. 10.3390/nano9081131 PMC6722545 31390794

[B118] Mahmoud N. N. Abu-Dahab R. Hamadneh L. A. Abuarqoub D. Jafar H. Khalil E. A. (2019a). Insights into the cellular uptake, cytotoxicity, and cellular death modality of phospholipid-coated gold nanorods toward breast cancer cell lines. Mol. Pharmaceutics. 16, 4149–4164. 10.1021/acs.molpharmaceut.9b00470 31398052

[B119] Manikandan M. Hasan N. Wu H.-F. (2013). Platinum nanoparticles for the photothermal treatment of Neuro 2A cancer cells. Biomaterials 34, 5833–5842. 10.1016/j.biomaterials.2013.03.077 23642996

[B120] Mbalaha Z. S. Edwards P. R. Birch D. J. S. Chen Y. (2019). Synthesis of small gold nanorods and their subsequent functionalization with hairpin single stranded DNA. ACS Omega. 4, 13740–13746. 10.1021/acsomega.9b01200 31497691 PMC6714599

[B121] Moreira A. F. Dias D. R. Costa E. C. Correia I. J. (2017). Thermo- and pH-responsive nano-in-micro particles for combinatorial drug delivery to cancer cells. Eur. J. Pharm. Sci. 104, 42–51. 10.1016/j.ejps.2017.03.033 28347775

[B122] Nabil M. Decuzzi P. Zunino P. (2015). Modelling mass and heat transfer in nano-based cancer hyperthermia. R Soc Open Sci. 2, 150447. 10.1098/rsos.150447 26587251 PMC4632523

[B123] Nair R. V. Santhakumar H. Jayasree R. S. (2018). Gold nanorods decorated with a cancer drug for multimodal imaging and therapy. Faraday Discuss. 207, 423–435. 10.1039/c7fd00185a 29355869

[B124] Nguyen T. M. Pettibone J. M. Gigault J. Hackley V. A. (2016). *In situ* monitoring, separation, and characterization of gold nanorod transformation during seed-mediated synthesis. Anal. Bioanal. Chem. 408, 2195–2201. 10.1007/s00216-016-9366-6 26873210 PMC13383878

[B125] Nguyen V. D. Min H.-K. Kim D.-H. Kim C.-S. Han J. Park J.-O. (2020). Macrophage-mediated delivery of multifunctional nanotherapeutics for synergistic chemo-photothermal therapy of solid tumors. ACS Appl. Mater. Inter. 12, 10130–10141. 10.1021/acsami.9b23632 32041404

[B126] Nikoobakht B. El-Sayed M. A. (2003). Preparation and growth mechanism of gold nanorods (NRs) using seed-mediated growth method. Chem. Mater. 15, 1957–1962. 10.1021/cm020732l

[B127] Ohta S. Glancy D. Chan W. C. W. (2016). DNA-controlled dynamic colloidal nanoparticle systems for mediating cellular interaction. Science 351, 841–845. 10.1126/science.aad4925 10.1126/science.aad4925 26912892

[B128] Olson E. S. Jiang T. Aguilera T. A. Nguyen Q. T. Ellies L. G. Scadeng M. (2010). Activatable cell penetrating peptides linked to nanoparticles as dual probes for *in vivo* fluorescence and MR imaging of proteases. Proc. Natl. Acad. Sci. 107, 4311–4316. 10.1073/pnas.0910283107 20160077 PMC2840175

[B129] Onaciu A. Braicu C. Zimta A.-A. Moldovan A. Stiufiuc R. Buse M. (2019). Gold nanorods: from anisotropy to opportunity. An evolution update. Nanomedicine 14, 1203–1226. 10.2217/nnm-2018-0409 31075049

[B130] Papaioannou L. Angelopoulou A. Hatziantoniou S. Papadimitriou M. Apostolou P. Papasotiriou I. (2018). Folic acid-functionalized gold nanorods for controlled paclitaxel delivery: in vitro evaluation and cell studies. AAPS PharmSciTech. 20, 13. 10.1208/s12249-018-1226-6 30560417

[B131] Parak W. J. (2016). Controlled interaction of nanoparticles with cells. Science. 351, 814–815. 10.1126/science.aaf0751 26912879

[B132] Park J. Choi Y. Chang H. Um W. Ryu J. H. Kwon I. C. (2019). Alliance with EPR effect: combined strategies to improve the EPR effect in the tumor microenvironment. Theranostics 9, 8073–8090. 10.7150/thno.37198 31754382 PMC6857053

[B133] Park K. Hsiao M.-s. Yi Y.-J. Izor S. Koerner H. Jawaid A. (2017). Highly concentrated seed-mediated synthesis of monodispersed gold nanorods. ACS Appl. Mater. Inter. 9, 26363–26371. 10.1021/acsami.7b08003 28714667

[B134] Patil S. Sandberg A. Heckert E. Self W. Seal S. (2007). Protein adsorption and cellular uptake of cerium oxide nanoparticles as a function of zeta potential. Biomaterials 28, 4600–4607. 10.1016/j.biomaterials.2007.07.029 17675227 PMC2259388

[B135] Patino T. Mahajan U. Palankar R. Medvedev N. Walowski J. Münzenberg M. (2015). Multifunctional gold nanorods for selective plasmonic photothermal therapy in pancreatic cancer cells using ultra-short pulse near-infrared laser irradiation. Nanoscale 7, 5328–5337. 10.1039/c5nr00114e 25721177

[B136] Peer D. Karp J. M. Hong S. Farokhzad O. C. Margalit R. Langer R. (2007). Nanocarriers as an emerging platform for cancer therapy. Nat. Nanotech 2, 751–760. 10.1038/nnano.2007.387 18654426

[B137] Peer D. Margalit R. (2004). Loading mitomycin C inside long circulating hyaluronan targeted nano-liposomes increases its antitumor activity in three mice tumor models. Int. J. Cancer 108, 780–789. 10.1002/ijc.11615 14696107

[B138] Piao J.-G. Gao F. Li Y. Yu L. Liu D. Tan Z.-B. (2018). pH-sensitive zwitterionic coating of gold nanocages improves tumor targeting and photothermal treatment efficacy. Nano Res. 11, 3193–3204. 10.1007/s12274-017-1736-7

[B139] Qiu Y. Liu Y. Wang L. Xu L. Bai R. Ji Y. (2010). Surface chemistry and aspect ratio mediated cellular uptake of Au nanorods. Biomaterials 31, 7606–7619. 10.1016/j.biomaterials.2010.06.051 20656344

[B140] Quan P. Bu W. Lin B. Jiang X. Wang L. (2019). Correlating ligand density with cellular uptake of gold nanorods revealed by X-ray reflectivity. J. Nanosci Nanotechnol. 19, 7557–7563. 10.1166/jnn.2019.16749 31196261

[B141] Rao L. Bu L.-L. Ma L. Wang W. Liu H. Wan D. (2018). Platelet-facilitated photothermal therapy of head and neck squamous cell carcinoma. Angew. Chem. Int. Ed. 57, 986–991. 10.1002/anie.201709457 29193651

[B142] Ratto F. (2014). Plasmonic particles that hit hypoxic cells. Adv. Funct. Mater. 25, 316-323. 10.1002/adfm.201402118

[B143] Ratto F. Matteini P. Rossi F. Pini R. (2010). Size and shape control in the overgrowth of gold nanorods. J. Nanopart Res. 12, 2029–2036. 10.1007/s11051-009-9712-0

[B144] Rayavarapu R. G. Ungureanu C. Krystek P. van Leeuwen T. G. Manohar S. (2010). Iodide impurities in hexadecyltrimethylammonium bromide (CTAB) products: lot−Lot variations and influence on gold nanorod synthesis. Langmuir. 26, 5050–5055. 10.1021/la100166f 20205463

[B145] Requejo K. I. Liopo A. V. Derry P. J. Zubarev E. R. (2017). Accelerating gold nanorod synthesis with nanomolar concentrations of poly(vinylpyrrolidone). Langmuir. 33, 12681–12688. 10.1021/acs.langmuir.7b02942 29032680

[B146] Requejo K. I. Liopo A. V. Zubarev E. R. (2020). Gold nanorod synthesis with small thiolated molecules. Langmuir. 36, 3758–3769. 10.1021/acs.langmuir.0c00302 32216357

[B147] Requejo K. I. Liopo A. V. Zubarev E. R. (2018) Synthesis of Gold Nanorods Using Poly(vinylpyrrolidone) of Different Molecular Weights as an Additive. ChemistrySelect. 3, 12192-12197. 10.1002/slct.201803337

[B148] Riley R. S. Day E. S. (2017). Gold nanoparticle-mediated photothermal therapy: applications and opportunities for multimodal cancer treatment. Wiley Interdiscip Rev Nanomed. Nanobiotechnol. 9. 10.1002/wnan.1449 PMC5474189 28160445

[B149] Roach L. Ye S. Moorcroft S. C. T. Critchley K. Coletta P. L. Evans S. D. (2018). Morphological control of seedlessly-synthesized gold nanorods using binary surfactants. Nanotechnology 29, 135601. 10.1088/1361-6528/aaa99d 29355832

[B150] Saha K. Kim S. T. Yan B. Miranda O. R. Alfonso F. S. Shlosman D. (2013). Surface functionality of nanoparticles determines cellular uptake mechanisms in mammalian cells. Small 9, 300–305. 10.1002/smll.201201129 22972519 PMC4070423

[B151] Sapareto S. A. Dewey W. C. (1984). Thermal dose determination in cancer therapy. Int. J. Radiat. Oncology*Biology*Physics 10, 787–800. 10.1016/0360-3016(84)90379-1 6547421

[B152] Sau T. K. Murphy C. J. (2004). Seeded high yield synthesis of short Au nanorods in aqueous solution. Langmuir. 20, 6414–6420. 10.1021/la049463z 15248731

[B153] Scarabelli L. Sánchez-Iglesias A. Pérez-Juste J. Liz-Marzán L. M. (2015). A "tips and tricks" practical guide to the synthesis of gold nanorods. J. Phys. Chem. Lett. 6, 4270–4279. 10.1021/acs.jpclett.5b02123 26538043

[B154] Seo B. Lim K. Kim S. S. Oh K. T. Lee E. S. Choi H.-G. (2019). Small gold nanorods-loaded hybrid albumin nanoparticles with high photothermal efficacy for tumor ablation. Colloids Surf. B: Biointerfaces 179, 340–351. 10.1016/j.colsurfb.2019.03.068 30991214

[B155] Si S. Leduc C. Delville M.-H. Lounis B. (2012). Short gold nanorod growth revisited: the critical role of the bromide counterion. Chemphyschem. 13, 193–202. 10.1002/cphc.201100710 22162413

[B156] Singh M. Harris-Birtill D. C. C. Zhou Y. Gallina M. E. Cass A. E. G. Hanna G. B. (2016). Application of gold nanorods for photothermal therapy in ex vivo human oesophagogastric adenocarcinoma. J Biomed. Nanotechnol. 12, 481–490. 10.1166/jbn.2016.2196 27280246

[B157] Song J. Pan J-B. Zhao W. Chen H-Y. Xu J-J. (2020). Gold nanorod-assisted near-infrared light-mediated regulation of membrane ion channels activates apoptotic pathways. Chem. Commun. (Camb). 56, 6118-6121. 10.1039/d0cc01858a 32364208

[B158] Song J. Yang X. Jacobson O. Huang P. Sun X. Lin L. (2015). Ultrasmall gold nanorod vesicles with enhanced tumor accumulation and fast excretion from the body for cancer therapy. Adv. Mater. 27, 4910–4917. 10.1002/adma.201502486 26198622

[B159] Su G. Yang C. Zhu J.-J. (2015). Fabrication of gold nanorods with tunable longitudinal surface plasmon resonance peaks by reductive dopamine. Langmuir. 31, 817–823. 10.1021/la504041f 25521416

[B160] Su S. Wang J. Vargas E. Wei J. Martínez-Zaguilán R. Sennoune S. R. (2016). Porphyrin immobilized nanographene oxide for enhanced and targeted photothermal therapy of brain cancer. ACS Biomater. Sci. Eng. 2, 1357–1366. 10.1021/acsbiomaterials.6b00290 33434989

[B161] Sun C.-Y. Liu Y. Du J.-Z. Cao Z.-T. Xu C.-F. Wang J. (2016). Facile generation of tumor-pH-labile linkage-bridged block copolymers for chemotherapeutic delivery. Angew. Chem. Int. Ed. 55, 1010–1014. 10.1002/anie.201509507 26756443

[B162] Sun Q. Shi X. Feng J. Zhang Q. Ao Z. Ji Y. (2018). Cytotoxicity and cellular responses of gold nanorods to smooth muscle cells dependent on surface chemistry coupled action. Small 14, e1803715. 10.1002/smll.201803715 30430733

[B163] Szychowski B. Leng H. Pelton M. Daniel M.-C. (2018). Controlled etching and tapering of Au nanorods using cysteamine. Nanoscale 10, 16830–16838. 10.1039/c8nr05325a 30167608

[B164] Takahata R. Yamazoe S. Koyasu K. Imura K. Tsukuda T. (2018). Gold ultrathin nanorods with controlled aspect ratios and surface modifications: formation mechanism and localized surface plasmon resonance. J. Am. Chem. Soc. 140, 6640–6647. 10.1021/jacs.8b02884 29694041

[B165] Tatini F. Landini I. Scaletti F. Massai L. Centi S. Ratto F. (2014). Size dependent biological profiles of PEGylated gold nanorods. J. Mater. Chem. B 2, 6072–6080. 10.1039/c4tb00991f 32261859

[B166] Tham H. P. Chen H. Tan Y. H. Qu Q. Sreejith S. Zhao L. (2016). Photosensitizer anchored gold nanorods for targeted combinational photothermal and photodynamic therapy. Chem. Commun. 52, 8854–8857. 10.1039/c6cc03076a 27346609

[B167] Tong W. Walsh M. J. Mulvaney P. Etheridge J. Funston A. M. (2017). Control of symmetry breaking size and aspect ratio in gold nanorods: underlying role of silver nitrate. J. Phys. Chem. C 121, 3549–3559. 10.1021/acs.jpcc.6b10343

[B168] Tong X. Wang Z. Sun X. Song J. Jacobson O. Niu G. (2016). Size dependent kinetics of gold nanorods in EPR mediated tumor delivery. Theranostics. 6, 2039–2051. 10.7150/thno.17098 27698939 PMC5039679

[B169] Vankayala R. Hwang K. C. (2018). Near-infrared-light-activatable nanomaterial-mediated phototheranostic nanomedicines: an emerging paradigm for cancer treatment. Adv. Mater. 30, 1706320. 10.1002/adma.201706320 29577458

[B170] Vigderman L. Zubarev E. R. (2013). High-yield synthesis of gold nanorods with longitudinal SPR peak greater than 1200 nm using hydroquinone as a reducing agent. Chem. Mater. 25, 1450–1457. 10.1021/cm303661d

[B171] Von Maltzahn G. Park J.-H. Agrawal A. Bandaru N. K. Das S. K. Sailor M. J. (2009). Computationally guided photothermal tumor therapy using long-circulating gold nanorod antennas. Cancer Res. 69, 3892–3900. 10.1158/0008-5472.can-08-4242 19366797 PMC2712876

[B172] Walsh M. J. Tong W. Katz-Boon H. Mulvaney P. Etheridge J. Funston A. M. (2017a). A mechanism for symmetry breaking and shape control in single-crystal gold nanorods. Acc. Chem. Res. 10.1021/acs.accounts.7b00313 29144733

[B173] Walsh M. J. Tong W. Katz-Boon H. Mulvaney P. Etheridge J. Funston A. M. (2017b). A mechanism for symmetry breaking and shape control in single-crystal gold nanorods. Acc. Chem. Res.. 10.1021/acs.accounts.7b00313 29144733

[B174] Wang L. Li D. Hao Y. Niu M. Hu Y. Zhao H. (2017). Gold nanorod–based poly(lactic-co-glycolic acid) with manganese dioxide core–shell structured multifunctional nanoplatform for cancer theranostic applications. Int J Nanomedicine Vol. 12, 3059–3075. 10.2147/ijn.s128844 PMC5399988 28450782

[B175] Wang S. Huang P. Chen X. (2016a). Stimuli-responsive programmed specific targeting in nanomedicine. ACS Nano. 10, 2991–2994. 10.1021/acsnano.6b00870 26881288 PMC5223089

[B176] Wang W. Li J. Lan S. Rong L. Liu Y. Sheng Y. (2016b). Seedless synthesis of gold nanorods using resveratrol as a reductant. Nanotechnology 27, 165601. 10.1088/0957-4484/27/16/165601 26954263

[B177] Wang Y. Guo Y. Shen Y. Chen R. Wang F. Zhou D. (2016c). HCl-retarded gold nanorod growth for aspect ratio and shape tuning. J. Nanosci Nanotechnol. 16, 1194–1201. 10.1166/jnn.2016.10637 27398586

[B178] Wang Y. Wang F. Liu Y. Xu S. Shen Y. Feng N. (2018). Glutathione detonated and pH responsive nano-clusters of Au nanorods with a high dose of DOX for treatment of multidrug resistant cancer. Acta Biomater. 75, 334–345. 10.1016/j.actbio.2018.06.012 29885528

[B179] Wirtz M. Yu S. Martin C. R. (2002). Template synthesized gold nanotube membranes for chemical separations and sensing. Analyst 127, 871–879. 10.1039/b201939f 12173641

[B180] Wistuba II Gelovani J. G. Jacoby J. J. Davis S. E. Herbst R. S. (2011). Methodological and practical challenges for personalized cancer therapies. Nat. Rev. Clin. Oncol. 8, 135–141. 10.1038/nrclinonc.2011.2 21364686

[B181] Wu L. Lin B. Yang H. Chen J. Mao Z. Wang W. (2019a). Enzyme-responsive multifunctional peptide coating of gold nanorods improves tumor targeting and photothermal therapy efficacy. Acta Biomater. 86, 363–372. 10.1016/j.actbio.2019.01.026 30660006

[B182] Wu Y. Ali M. R. K. Dong B. Han T. Chen K. Chen J. (2018). Gold nanorod photothermal therapy alters cell junctions and actin network in inhibiting cancer cell collective migration. ACS nano. 12, 9279–9290. 10.1021/acsnano.8b04128 30118603 PMC6156989

[B183] Wu Z. Liang Y. Cao L. Guo Q. Jiang S. Mao F. (2019b). High-yield synthesis of monodisperse gold nanorods with a tunable plasmon wavelength using 3-aminophenol as the reducing agent. Nanoscale 11, 22890–22898. 10.1039/c9nr07949a 31763638

[B184] Xia K. Zhang L. Huang Y. Lu Z. (2015). Preparation of gold nanorods and their applications in photothermal therapy. J. Nanosci Nanotechnol. 15, 63–73. 10.1166/jnn.2015.9586 26328306

[B185] Xu C. Zhang T. Lu G. Chen K. Tao J. Zhang Y. (2020). Disulfiram-gold-nanorod integrate for effective tumor targeting and photothermal-chemical synergistic therapy. Biomater. Sci. 8, 3310–3319. 10.1039/d0bm00062k 32400782

[B186] Xu J.-Q. Duo H.-H. Zhang Y.-G. Zhang X.-W. Fang W. Liu Y.-L. (2016). Photochemical synthesis of shape-controlled nanostructured gold on zinc oxide nanorods as photocatalytically renewable sensors. Anal. Chem. 88, 3789–3795. 10.1021/acs.analchem.5b04810 26928162

[B187] Xu W. Qian J. Hou G. Suo A. Wang Y. Wang J. (2017). Hyaluronic acid-functionalized gold nanorods with pH/NIR dual-responsive drug release for synergetic targeted photothermal chemotherapy of breast cancer. ACS Appl. Mater. Inter. 9, 36533–36547. 10.1021/acsami.7b08700 28975790

[B188] Xu W. Qian J. Hou G. Wang Y. Wang J. Sun T. (2019). A dual-targeted hyaluronic acid-gold nanorod platform with triple-stimuli responsiveness for photodynamic/photothermal therapy of breast cancer. Acta Biomater. 83, 400–413. 10.1016/j.actbio.2018.11.026 30465921

[B189] Yan C. Wang Y. Tian Q. Wu H. Yang S. (2018). Concentration effect on large scale synthesis of high quality small gold nanorods and their potential role in cancer theranostics. Mater. Sci. Eng. C. 87, 120–127. 10.1016/j.msec.2018.02.021 29549941

[B190] Yang H. Chen Z. Zhang L. Yung W.-Y. Leung K. C.-F. Chan H. Y. E. (2016a). Mechanism for the cellular uptake of targeted gold nanorods of defined aspect ratios. Small 12, 5178–5189. 10.1002/smll.201601483 27442290

[B191] Yang J. Choi J. Bang D. Kim E. Lim E.-K. Park H. (2011). Convertible organic nanoparticles for near-infrared photothermal ablation of cancer cells. Angew. Chem. Int. Ed. 50, 441–444. 10.1002/anie.201005075 21132823

[B192] Yang M. Liu Y. Hou W. Zhi X. Zhang C. Jiang X. (2017). Mitomycin C-treated human-induced pluripotent stem cells as a safe delivery system of gold nanorods for targeted photothermal therapy of gastric cancer. Nanoscale 9, 334–340. 10.1039/c6nr06851k 27922138

[B193] Yang Y. Zhang J. Xia F. Zhang C. Qian Q. Zhi X. (2016b). Human CIK cells loaded with Au nanorods as a theranostic platform for targeted photoacoustic imaging and enhanced immunotherapy and photothermal therapy. Nanoscale Res. Lett. 11, 285. 10.1186/s11671-016-1468-8 27271853 PMC4894853

[B194] Ye L. Mosbach K. (2008). Molecular imprinting: synthetic materials as substitutes for biological antibodies and receptors†. Chem. Mater. 20, 859–868. 10.1021/cm703190w

[B195] Yin D. Li X. Ma Y. Liu Z. (2017). Targeted cancer imaging and photothermal therapy via monosaccharide-imprinted gold nanorods. Chem. Commun. 53, 6716–6719. 10.1039/c7cc02247f 28585650

[B196] Young J. K. Figueroa E. R. Drezek R. A. (2012). Tunable nanostructures as photothermal theranostic agents. Ann. Biomed. Eng. 40, 438–459. 10.1007/s10439-011-0472-5 22134466

[B197] Zarska M. Sramek M. Novotny F. Havel F. Babelova A. Mrazkova B. (2018). Biological safety and tissue distribution of (16-mercaptohexadecyl)trimethylammonium bromide-modified cationic gold nanorods. Biomaterials 154, 275–290. 10.1016/j.biomaterials.2017.10.044 29149721

[B198] Zhang C. Cheng X. Chen M. Sheng J. Ren J. Jiang Z. (2017a). Fluorescence guided photothermal/photodynamic ablation of tumours using pH-responsive chlorin e6-conjugated gold nanorods. Colloids Surf. B: Biointerfaces 160, 345–354. 10.1016/j.colsurfb.2017.09.045 28961542

[B199] Zhang J. Feng Y. Mi J. Shen Y. Tu Z. Liu L. (2018). Photothermal lysis of pathogenic bacteria by platinum nanodots decorated gold nanorods under near infrared irradiation. J. Hazard. Mater. 342, 121–130. 10.1016/j.jhazmat.2017.07.053 28826054

[B200] Zhang J. Wang M. Webster T. J. (2017b). Growth process and anticancer properties of gold nanorods. J. Biomed. Mater. Res. 105, 2616–2621. 10.1002/jbm.a.36119 28544392

[B201] Zhang L. Xia K. Bai Y.-Y. Lu Z. Tang Y. Deng Y. (2014a). Synthesis of gold nanorods and their functionalization with bovine serum albumin for optical hyperthermia. J. Biomed. Nanotechnol. 10, 1440–1449. 10.1166/jbn.2014.1932 25016644

[B202] Zhang M. Kim H. S. Jin T. Moon W. K. (2017c). Near-infrared photothermal therapy using EGFR-targeted gold nanoparticles increases autophagic cell death in breast cancer. J. Photochem. Photobiol. B: Biol. 170, 58–64. 10.1016/j.jphotobiol.2017.03.025 28390259

[B203] Zhang W. Yu W. Ding X. Yin C. Yan J. Yang E. (2019). Self-assembled thermal gold nanorod-loaded thermosensitive liposome-encapsulated ganoderic acid for antibacterial and cancer photochemotherapy. Artif. Cell Nanomedicine, Biotechnol. 47, 406–419. 10.1080/21691401.2018.1559177 30724609

[B204] Zhang Y. Feng Y. Huang Y. Wang Y. Qiu L. Liu Y. (2020). Tumor-targeted gene silencing Ido synergizes PTT-induced apoptosis and enhances anti-tumor immunity. Front. Immunol. 11, 968. 10.3389/fimmu.2020.00968 32582152 PMC7295913

[B205] Zhang Y. Wei G. Yu J. Birch D. J. S. Chen Y. (2015). Surface plasmon enhanced energy transfer between gold nanorods and fluorophores: application to endocytosis study and RNA detection. Faraday Discuss. 178, 383–394. 10.1039/c4fd00199k 25778775

[B206] Zhang Z. Wang J. Nie X. Wen T. Ji Y. Wu X. (2014b). Near infrared laser-induced targeted cancer therapy using thermoresponsive polymer encapsulated gold nanorods. J. Am. Chem. Soc. 136, 7317–7326. 10.1021/ja412735p 24773323

[B207] Zhen X. Cheng P. Pu K. (2019). Recent advances in cell membrane-camouflaged nanoparticles for cancer phototherapy. Small. 15, e1804105. 10.1002/smll.201804105 30457701

[B208] Zheng X. Xing D. Zhou F. Wu B. Chen W. R. (2011). Indocyanine green-containing nanostructure as near infrared dual-functional targeting probes for optical imaging and photothermal therapy. Mol. Pharmaceutics 8, 447–456. 10.1021/mp100301t 21197955

[B209] Zhou J. Liu Z. Li F. (2012). Upconversion nanophosphors for small-animal imaging. Chem. Soc. Rev. 41, 1323–1349. 10.1039/c1cs15187h 22008740

[B210] Zhu H. Chen Y. Yan F.-J. Chen J. Tao X.-F. Ling J. (2017). Polysarcosine brush stabilized gold nanorods for *in vivo* near-infrared photothermal tumor therapy. Acta Biomater. 50, 534–545. 10.1016/j.actbio.2016.12.050 28027959

[B211] Zhu L. Wang T. Perche F. Taigind A. Torchilin V. P. (2013). Enhanced anticancer activity of nanopreparation containing an MMP2-sensitive PEG-drug conjugate and cell-penetrating moiety. Proc. Natl. Acad. Sci. 110, 17047–17052. 10.1073/pnas.1304987110 24062440 PMC3801051

[B212] Zhu X.-M. Fang C. Jia H. Huang Y. Cheng C. H. K. Ko C.-H. (2014). Cellular uptake behaviour, photothermal therapy performance, and cytotoxicity of gold nanorods with various coatings. Nanoscale 6, 11462–11472. 10.1039/c4nr03865g 25155843

